# Increasing PFAS concentrations in human serum correlate with elevated blood lipid levels

**DOI:** 10.1039/d5va00483g

**Published:** 2026-02-16

**Authors:** Ashlee T. Falls, Anna K. Boatman, Jack P. Ryan, Amie M. Solosky, James N. Dodds, Jessie R. Chappel, Allison N. Fry, Kaylie I. Kirkwood-Donelson, Heather M. Stapleton, Erin S. Baker

**Affiliations:** a Department of Chemistry, University of North Carolina at Chapel Hill Chapel Hill NC 27514 USA erinmsb@unc.edu; b Nicholas School of the Environment, Duke University Durham NC 27708 USA heather.stapleton@duke.edu; c Bioinformatics Research Center, Department of Biological Sciences, North Carolina State University Raleigh NC 27606 USA; d National Institute of Environmental Health Sciences Durham NC 27709 USA

## Abstract

Per- and polyfluoroalkyl substances (PFAS) are a large group of synthetic chemicals which have been detected in the blood of >99% people worldwide. Currently, certain PFAS are linked to elevated cholesterol levels in humans, but few studies have assessed changes in specific lipid species to assess mechanistic changes. In this study, 78 serum samples were attained from 49 participants exposed to elevated PFAS through drinking water and 29 occupationally exposed firefighters. PFAS serum concentrations were initially assessed, and drinking water exposure participants illustrated higher PFAS serum levels than both the firefighters and national median values from the National Health and Examination Survey (NHANES). Participants were then regrouped for lipidomic analyses using their summed serum concentration for 7 PFAS (Σ7 PFAS). Thirty-four participants in our study had Σ7 PFAS concentrations ≥20 ng mL^−1^, a level that has been associated with increased risk of dyslipidemia, thyroid dysfunction and cancer according to the National Academies PFAS Exposure Guidance Report. Statistical analyses revealed that 24 lipids out of 387 detected in all participants were significantly higher in participants with Σ7 PFAS values ≥20 ng mL^−1^. Triglycerides and phosphatidylethanolamines specifically represented 62.5% of these 24 lipids, suggesting alteration of cellular membrane structures and energy storage. A statistical assessment on the female-only samples from the drinking water cohort was also performed to reduce bias due to sex, age and occupational covariates and further validated these trends. This study therefore illustrates increased serum PFAS concentrations correlate with elevated lipid species and molecular pathway alterations in highly exposed individuals.

## Abstract 2

Increased serum PFAS concentrations correlate with elevated lipid species in exposed individuals, suggesting monitoring for adverse health effects is necessary.

## Graphical abstract

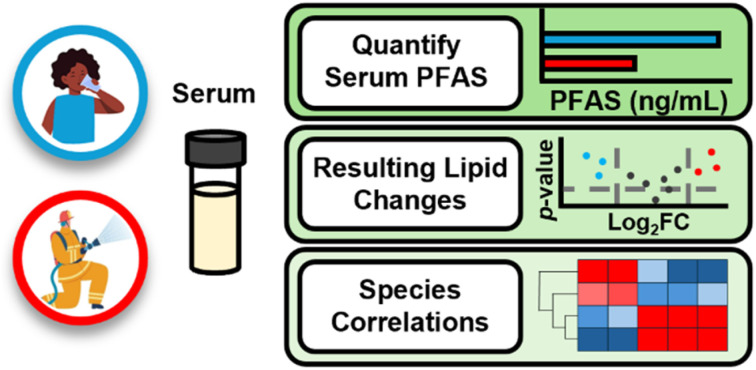

**Environmental significance**

Per- and polyfluoroalkyl substances (PFAS) are a large group of synthetic chemicals that have polluted global water supplies for decades, and PFAS have been detected in the blood of >99% of people worldwide. However, while current studies have linked PFAS to elevated cholesterol levels in humans, few studies have assessed changes in specific lipid species to assess mechanistic changes. In this study, individuals with known PFAS-contaminated drinking water sources were found to have ≥20 ng mL^−1^ of Σ7 serum PFAS levels, a level stated by the National Academies PFAS Exposure Guidance Report as concern for dyslipidemia. These individuals also had increased triglycerides and phosphatidylethanolamines compared to lesser-exposed humans, revealing key molecular pathway alterations in relation to heightened PFAS exposures.

## 1 Introduction

Per- and polyfluoroalkyl substances (PFAS) are a large class of synthetic organofluorine chemicals incorporated in manufacturing processes and consumer products since the 1940s for their thermal stability, resistance to degradation, and oil- and water-resistance.^1^ While there are multiple definitions of PFAS, the Organisation for Economic Co-operation and Development's (OECD) definition broadly defines PFAS as any molecule having one fully fluorinated methyl (–CF_3_) or methylene (–CF_2_–) carbon atom.^2,3^ To date, this definition includes more than 7 million chemicals from the PubChem library and encompasses PFAS with a wide variety of chemical structures and properties.^2,4,5^ For example, some PFAS, such as perfluorooctanoic acid (PFOA) and perfluorooctane sulfonic acid (PFOS), are known to bioaccumulate in the human body and are linked to an array of health concerns such as low birth weight, weakened immune responses, cancer, and elevated cholesterol levels.^6^ Despite these potential health effects, PFAS are commonly used in a wide variety of consumer and industrial products such as aqueous film-forming foams (AFFFs), nonstick cookware, cosmetics, and food packaging due to their unique properties.^7–12^ PFAS are also frequently discharged into the environment, with major sources including manufacturing waste, landfill leachate, and the application of AFFFs for extinguishing hydrocarbon-fuel-based fires and use by the military and civilian fire departments during controlled Class B, or oil-based, fire safety trainings and active burn scenes.^13–18^ While PFAS levels in blood serum are generally considered the gold standard for monitoring exposure, the routes of exposures as well as the magnitude of exposures vary greatly and drinking water and occupational exposures are of particular concern and are the focus of this study.^19–21^

Drinking water exposure to PFAS represents an area of great concern for human health. In April 2024, the United States Environmental Protection Agency (U.S. EPA) announced the PFAS National Primary Drinking Water Regulation (NPDWR) which they noted would establish limits for six PFAS, prevent drinking water exposure for approximately 100 million people, and reduce PFAS-attributable illnesses like thyroid, kidney, or liver disease.^22^ The specific PFAS listed in the NPDWR include PFOA, PFOS, perfluorohexane sulfonic acid (PFHxS), perfluorononanoic acid (PFNA), hexafluoropropylene oxide dimer acid (HFPO-DA or GenX), and mixtures containing two or more of PFHxS, PFNA, GenX, and perfluorobutane sulfonic acid (PFBS).^22^ The NPDWR also gave public water municipalities until 2027 to complete initial monitoring and 2029 to implement solutions to reduce PFAS if the initial results exceed the current enforceable maximum contaminant levels (MCLs). However, despite these intended regulations, the U.S. EPA announced in May 2025 that MCLs for only PFOS and PFOA will be kept, and the remaining four PFAS and mixtures will be eliminated from regulation.^23,24^ They also noted that compliance deadlines will be extended. While this regulation may result in decreased exposure for these 2 PFAS, there are over 7 million other known PFAS structures that remain unregulated, many with unknown health effects.^4,25^ Therefore, it is imperative to increase our understanding of health effects and molecular changes occurring in individuals exposed to these and other PFAS.^4,25,26^

Occupational PFAS exposure is also an area of great concern, particularly for firefighters. Several studies have found that firefighters have higher blood PFAS levels compared to the general population.^27–31^ While the exact source of this higher exposure is unclear, there are some suggestions that historic use and training with AFFFs may be responsible.^12,32^ Exposure to soot and smoke during firefighting activities may also lead to exposure to PFAS given their use in building materials and furnishings. In addition, firefighter turnout gear has been shown to contain PFAS treatments to provide water repellency, particularly in the outer shell; however, it is unknown whether the use of PFAS in gear has led to significant exposure among firefighters.^12,21,33,34^ In 2022, the International Agency for Research on Cancer (IARC) changed firefighting occupational exposure from “Group 2B” carcinogen (possibly carcinogenic to humans) to a “Group 1” (carcinogenic to humans) due to a variety of exposures (*e.g.*, PFAS, particulate matter, polycyclic aromatic hydrocarbons (PAHs), volatile organic compounds (VOCs), and asbestos), thus putting firefighting exposure on par with exposure to tobacco, benzene, and PFOA.^21,35–40^ Moreover, studies have shown linkages between PFAS exposure and a variety of cancers including kidney cancer, prostate and testicular cancer, and non-Hodgkin's lymphoma, several of which are cancer types that firefighters have increased risk of developing compared to the general public.^21,41,42^ It is therefore crucial to test and monitor firefighters for PFAS exposure and biological responses.

Lipids are involved in a variety of cellular processes, such as cell signaling, communication, and energy storage.^43,44^ Evaluating changes in a broad variety of lipid species can provide insights into the impacts of chemical exposure on biological functions. This study therefore sought to interrogate human blood serum for PFAS and conduct a lipidomic analyses to assess associations. Currently, >50 000 known lipid species spanning eight different categories are listed in LIPID MAPS, yet routine blood lipid panels evaluate total triglycerides and cholesterol levels only.^45^ Recent studies have revealed that triglycerides are positively associated with PFOA, PFNA, perfluoroheptanoic acid (PFHpA), and perfluorodecanoic acid (PFDA).^46^ Additional work has also found that serum PFOA and PFHxS are linked to increased total cholesterol and non-high-density lipoprotein (non-HDL) cholesterol.^19^ Thus, a more comprehensive lipidomic analysis on individuals exposed to PFAS will provide greater insight into lipidomic differences and concerns for those with high PFAS serum levels.^44^

## 2 Materials and methods

### 2.1 Study cohorts and serum collection

Two cohorts, subsequently referred to as the Drinking Water Exposure (DW) and Firefighter Exposure (FF) cohorts, were evaluated in this study and named for their primary suspected PFAS exposure routes. For the DW cohort, between Fall 2019 and Summer 2020, individuals living in Pittsboro, North Carolina (NC) were invited to participate in a study to evaluate PFAS in their drinking water and in their blood. Further details on recruitment and sample collection are described in Hall *et al.* 2023.^19^ In a separate study conducted between 2022–2023, for the FF cohort, City of Durham, NC firefighters were invited to participate in a research study examining their exposure to a range of contaminants, including PFAS. Further details on this study, including recruitment and sample collection, are described in more detail in Hoxie *et al.* 2025.^20,47^ The Duke University Institutional Review Board reviewed and approved all study protocols prior to recruitment, and all study participants provided informed consent.

### 2.2 Experimental methods

For the 78 human serum samples collected, both PFAS targeted and suspect screening analyses (SSA) were performed to provide absolute concentrations for 13 PFAS and evaluate the presence of 126 other possible PFAS. Suspect screening of 877 lipids was also performed to understand lipidomic differences in the participants studied. The PFAS and lipidomic evaluations involved three unique extraction methods and three unique instrumental analyses. In brief, for the targeted PFAS analyses, serum samples were extracted by solid phase extraction (SPE) and analyzed *via* LC-MS/MS using an inclusion list of 34 (FFs) or 19 (DWs) different PFAS. For PFAS SSA, serum samples were extracted in acetonitrile and analyzed *via* liquid chromatography-ion mobility spectrometry-mass spectrometry (LC-IMS-MS). For lipidomic SSA, serum samples were extracted using a modified-Folch lipid extraction and analyzed on the same platform as the PFAS SSA.^48^ Detailed experimental methods for the targeted PFAS analyses are provided in Hall *et al.* 2023 and Hoxie *et al.* 2025.^19,20,47^ The extraction methods for PFAS and lipidomic SSA are provided in SI. Additionally, the instrumental methods for the PFAS and lipidomic SSA are provided in Tables S1–S8. This information includes chemicals used (Table S1), PFAS and lipidomic SSA instrumentation methods including liquid chromatography (LC) gradients (Tables S2 and S5), electrospray ionization (ESI) source conditions (Tables S3 and S6), ion mobility spectrometry-mass spectrometry settings (IMS-MS) (Tables S4 and S8), and collision induced dissociation (CID) energy ramp method for the lipidomic analysis (Table S7).

### 2.3 PFAS targeted identification and statistical analysis approaches

In the previous targeted studies, 19 PFAS were quantified in the DW study and 34 were quantified in the FF study; however, only 13 PFAS were quantified in both groups (Table S9).^19,20,47^ The sums for these 13 PFAS were calculated and a Wilcoxon rank-sum test with Bonferroni corrections was performed using RStudio (v2024.04.2) to assess differences among groups. The National Health and Nutrition Examination Survey (NHANES) of 2017–2018 reports the concentration values for 5 of these PFAS in 1929 individuals as a reflection of the U.S. national median serum PFAS concentrations: PFOA, PFNA, PFDA, PFHxS, and PFOS.^49^ To compare the exposure groups to these national values and to each other, differences in median concentration values were assessed using a Kruskal–Wallis test with a Benjamini–Hochberg-adjusted Pairwise Wilcoxon rank-sum test. The sum of the 5 PFAS concentrations included in the NHANES analysis, as well as perfluoroundecanoic acid (PFUdA) and methyl perfluorooctane sulfonamido acetic acid (MeFOSAA), was calculated for each sample to further inform lipidomic analyses. These Σ7 PFAS were selected according to the National Academies guideline that individuals with serum PFAS concentrations of 20 ng mL^−1^ or higher (NHANES 5 PFAS + PFUdA + MeFOSAA) should be encouraged by clinicians to reduce PFAS exposure and be prioritized for dyslipidemia screening, thyroid function testing, kidney and testicular cancer screening, and ulcerative colitis testing.^50^ Because MeFOSAA was not quantified in all samples, it was reported as 0 ng mL^−1^ for all samples for consistency.

### 2.4 PFAS SSA identification and statistical analysis approaches

While targeted analyses are useful for quantitative evaluations of PFAS, they rely on the availability of standards and often fail to capture other analytes that may be in the sample. Non-targeted analyses (NTA) are useful approaches that allow researchers to confidently identify PFAS based on accurate mass measurements and other characteristics of PFAS, thus increasing the number of PFAS identifications and information for future studies.^51,52^ Suspect screening allows NTA data to be compared to known PFAS libraries to see if any detected NTA features are already known to exist. Thus, to complement the targeted information, SSA were performed on the same samples using a LC-IMS-MS platform to identify additional PFAS in the serum samples. 126 PFAS from an in-house library were assessed for their presence and relative abundance in the two cohorts to understand if certain PFAS are more prevalent in one cohort.^53^ In brief, PFAS detections were based on matching LC retention time (RT) within the conservative integration band of one minute of in-house library standards, as RTs have been observed to shift beyond the library entries in different matrices (*e.g.*, solvent *versus* serum). Also, for consistent integrations between samples, some PFAS examined in this work consist of both branched and linear variants which also have shifted RTs (Table S10). Identifications were furthermore based on collision cross section (CCS) values based on drift times within the resolving power window of 30, and mass-to-charge ratios (*m*/*z*) with mass errors ≤10 ppm to obtain feature identifications at a confidence level of 1 confirmed by reference standards analyzed under identical conditions.^54^ Peak areas were ratioed to the abundance of their corresponding internal standard when exact matches were available by a light/heavy (^12^C/^13^C) ratio to obtain normalized peak areas, or relative abundances. When an exact match PFAS standard was not available, a surrogate standard was assigned based on similarities of class, chain length, and retention time (Table S10). Relative PFAS abundances were calculated by subtracting the average of the method blank peak areas from the normalized peak area ratios. The limit of detection (LOD) was set as the average detection in the method blanks plus 3 times the standard deviation. Positive values following subtraction less than the LOD were replaced with the LOD/2 and values that were negative following the subtraction were set to zero.^19^ Results were imported into RStudio (v2024.04.2) for a Wilcoxon rank-sum test with Bonferroni *p*-value corrections for group differences for PFAS not previously targeted and detected in at least 3 samples in both groups.^55,56^

### 2.5 Lipid SSA identification and statistical analysis approaches

Lipidomic SSA were also performed on all samples for 877 lipids included in our in-house lipid library.^57^ Peaks were selected based on RT, CCS, and *m*/*z* as previously described, as well as fragmentation patterns for 387 lipid species found in all samples. The corresponding peak areas were log_2_ transformed for normalization to evaluate endogenous molecular changes due to PFAS serum levels. Additional annotation details are included in the Lipid Reporting Checklist in the SI. For the assessment of lipid differences due to high PFAS serum levels, all 78 samples were split into categories of ≥20 ng mL^−1^ and <20 ng mL^−1^ based on the Σ7 values from the National Academies guidelines previously described.^50^ Of note, the National Academies provides clinical guidance for treating individuals with Σ7 PFAS serum levels between 2 and 20 ng mL^−1^, particularly pregnant individuals, whereas individuals with <2 ng mL^−1^ should receive the usual standard of care. However, no individual in this study had levels below 2 ng mL^−1^, therefore all samples <20 ng mL^−1^ were grouped. A Mann–Whitney *U* test was performed with Benjamini–Hochberg *p*-value corrections to identify statistically significant lipids and fold change comparisons between ≥20 ng mL^−1^ and <20 ng mL^−1^ exposure groups. To determine if Σ7 PFAS concentrations, along with covariates (sex and age), may be predictors of lipid abundance, a linear mixed-effects model was applied with a random effect to account for non-independence between lipid abundances from the same individual. Statistical analyses were performed using RStudio (v2024.04.2).

### 2.6 PFAS and lipid integration

PFAS SSA peak areas and lipid SSA peak areas were imported into MetaboAnalyst 6.0 separately for data visualization by principal component analysis (PCA) to observe separations between the ≥20 ng mL^−1^ and <20 ng mL^−1^ exposure groups.^58^ Additionally, further data visualization for the lipids was performed using hierarchical clustering (based on Euclidean distances and clustering with the Ward method) to see how lipid abundances trend between the two exposure groups. All lipid features were imported into RStudio (v2024.04.2) with PFAS peak areas of 8 PFAS detected in ≥50% of samples for Spearman correlation tests to assess significant relationships between analytes.

## 3 Results and discussion

**Fig. 1:**
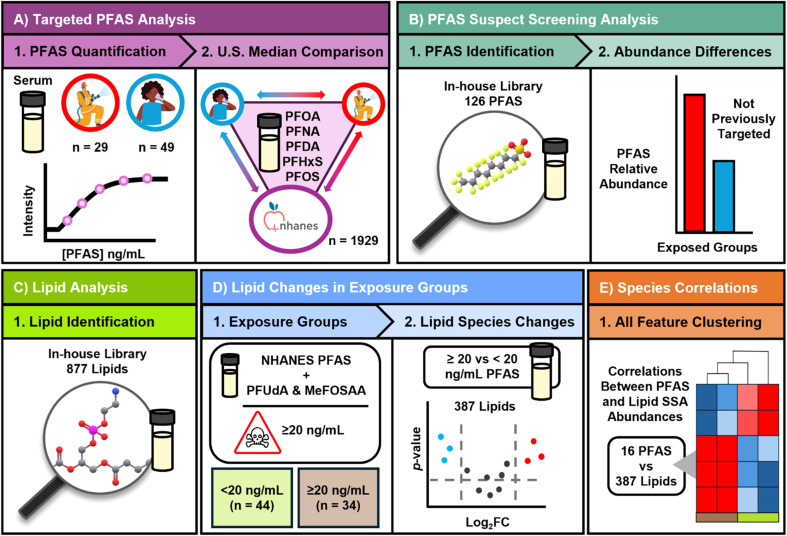
The workflow for analyses performed in this study. (A.1) Initially, 13 PFAS were quantified in the 78 human serum samples for two PFAS exposed cohorts: firefighter occupational exposure (*n* = 29, red) and drinking water exposure (*n* = 49, blue). (A.2) Five of the 13 quantified PFAS have been reported by NHANES (*n* = 1929) as median American serum concentrations, including PFOA, PFNA, PFDA, PFHxS, and PFOS. This information was used to compare exposed groups to the national medians. (B.1) PFAS suspect screening analyses were performed to identify unique PFAS species beyond the targeted analytes. (B.2) The relative abundances of the SSA PFAS were compared between exposed cohorts. (C) Lipid suspect screening analyses were performed. (D.1) For 7 PFAS, including the 5 NHANES PFAS plus PFUdA and MeFOSAA (Σ7), National Academies reports that participants with serum levels ≥20 ng mL^−1^ are at risk for health complications. Participants were regrouped based on their Σ7 totals for risk assessment: ≥20 ng mL^−1^ (*n* = 34) or <20 ng mL^−1^ (*n* = 44). (D.2) Using the established ≥20 ng mL^−1^ and <20 ng mL^−1^ PFAS groups, 387 lipid species were assessed for abundance changes. (E) Unsupervised hierarchical clustering was then performed on all detected PFAS and lipid species abundances to determine correlations and sample clustering.

In this study, we were interested in assessing whether correlations exist between serum PFAS levels and endogenous molecules, as previous work has shown correlations between PFAS exposure and increases in certain lipid levels.^44,59^ Therefore, we used samples from our drinking water (DW) and firefighter (FF) exposure cohorts to assess PFAS levels and then lipidomic changes. Specifically, the serum from DW individuals in the town of Pittsboro, NC were assessed in this study. Pittsboro has previously been documented as having PFAS contaminated drinking water sourced from the Haw River and Jordan Lake due to discharge by upstream industrial wastewater facilities.^19,60^ In contrast, a cohort of firefighters from Durham County, NC have occupational exposures to PFAS as previously described, but their drinking water supplies are not excessively contaminated with PFAS and have been closely monitored since 2018.^21,61^ These firefighters also live in a similar area of NC to those in the DW cohort (although they have different drinking water sources), so the groups have similar day-to-day environments. Previous work from Hall *et al.* 2023 and Hoxie *et al.* 2025 provided quantitative serum PFAS for the DW cohort (*n* = 49) and FF cohort (*n* = 29) in separate studies; however, here we wanted to assess both studies together to understand how the PFAS serum concentrations from the targeted PFAS studies compared.^19,20,47^ We also performed SSA on the serum from both cohorts to evaluate the presence and changes in 126 additional PFAS and 877 lipids. An overview of the study workflow is provided in Fig. 1.

### 3.1 Targeted PFAS analysis

**Fig. 2:**
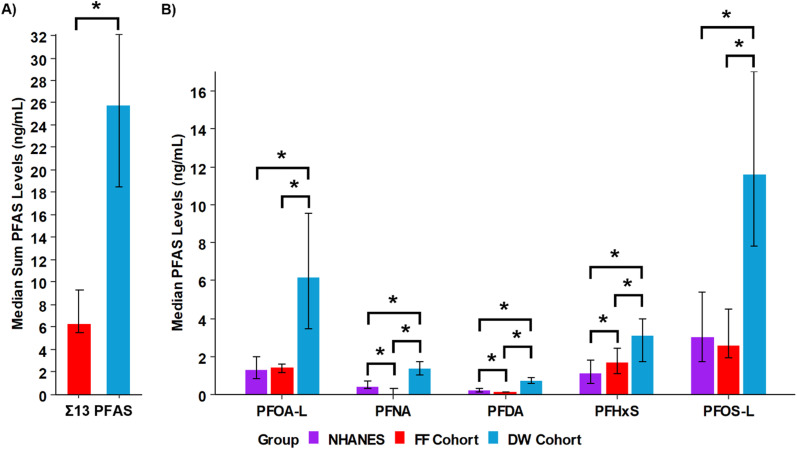
PFAS concentration comparisons between the firefighter, drinking water and NHANES cohorts. (A) The sum of 13 PFAS concentrations from the targeted study are shown for the firefighters (red) *versus* the drinking water group (blue) and are statistically significant, denoted by a single asterisk (*) (Wilcoxon rank-sum test, Bonferroni; *p*_adj_ < 0.05). (B) The medians are shown for 5 PFAS from NHANES (purple) in the bar charts, and statistically significant values are denoted a single asterisk (*) (Kruskal–Wallis, Benjamini–Hochberg-adjusted Pairwise Wilcoxon rank-sum test; *p*_adj_ < 0.05). Error bars represent the 25th and 75th percentiles.

In the targeted studies for both the DW and FF cohorts, a total of 13 PFAS were previously quantified (Table S9) and are utilized in the analyses herein.^19,20,47^ To understand the PFAS exposure for both cohorts, all 13 PFAS were initially summed (Σ13 PFAS). Interestingly, the median Σ13 PFAS value for the DW cohort was more than 4× higher than the FF cohort (25.7 ng mL^−1^*versus* 6.3 ng mL^−1^) and statistically significant (Wilcoxon rank-sum test, Bonferroni; *p*_adj_ < 0.05) (Fig. 2A). To further understand how the PFAS levels compare to the national median, data from the 2017–2018 National Health and Nutrition Examination Survey (NHANES) was used (Fig. 2B). In this survey, 1929 people were evaluated and the median values (25th percentile, 75th percentile) for 5 individual PFAS were reported including: linear perfluorooctanoic acid (L-PFOA, 1.3 (0.8, 2.0) ng mL^−1^), perfluorononanoic acid (PFNA, 0.4 (0.3, 0.7) ng mL^−1^), perfluorodecanoic acid (PFDA, 0.2 (0.1, 0.3) ng mL^−1^), perfluorohexane sulfonic acid (PFHxS, 1.1 (0.6, 1.8) ng mL^−1^), and linear perfluorooctane sulfonic acid (L-PFOS, 3.0 (1.7, 5.4) ng mL^−1^). A comparison of the NHANES values to our cohorts illustrated that the FF cohort was statistically higher for PFHxS, but lower for PFNA and PFDA (Fig. 2B and Table S11). However, all 5 PFAS were statistically higher in the DW cohort compared to both the NHANES and the FF cohort. While we had expected to see higher PFAS levels in the FF cohort due to their occupational exposure, this comparison suggests that drinking water could be a more potent exposure route of the PFAS included in the analysis for communities in this area.^60,62–67^ However, because these targeted results do not account for all possible PFAS, SSA were also performed to increase our comprehensiveness of unique and additional existing exposures.

### 3.2 PFAS suspect screening analysis

**Fig. 3:**
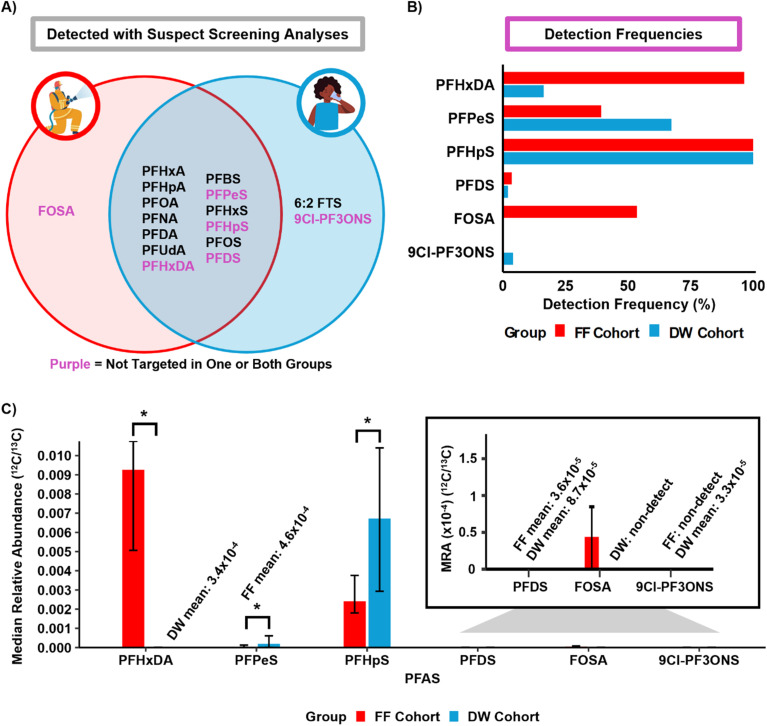
PFAS detections and comparisons in the firefighter and drinking water cohorts. (A) Venn diagrams of the 16 PFAS detected using suspect screening analyses. PFAS that were detected with suspect screening analyses that were not previously targeted in one or both groups are shown in purple. (B) The detection frequency of the 6 PFAS found with SSA for each exposure group. (C) The median relative abundances (MRA) obtained by ^12^C/^13^C peak area normalization for the 6 PFAS found from suspect screening illustrate that the firefighters had a higher level of PFHxDA (Wilcoxon rank-sum test, Bonferroni; *p*_adj_ < 0.05) while the drinking water cohort had more PFPeS and PFHpS. Statistical significance is denoted by a single asterisk (*). Statistical testing for the remaining 3 PFAS was not applicable due to low or 0% detection frequencies. For FF and DW median values that were 0, the mean values are displayed to differentiate medians of 0 *versus* non-detects.

To further assess the extent of PFAS exposure to these individuals, we leveraged LC-IMS-MS separations to perform SSA using an in-house library of 126 PFAS.^53^ In total, 16 PFAS were identified in the serum for the participant samples, including 10 from the targeted study. Six new PFAS were detected in the participants, and the 3 from the targeted study not observed with SSA were at very low concentrations due to differences in extraction methods and low abundance analytes not being picked up by the different instrument used for SSA. Of the 16 PFAS detected with SSA, 13 were detected in at least one sample from both cohorts, including 6 perfluoroalkyl sulfonic acids (C4–C8, and C10 PFSAs) and 7 perfluoroalkyl carboxylic acids (C6–C11 and C16 PFCAs) (Fig. 3A). For the other 3 SSA PFAS, perfluorooctane sulfonamide (FOSA, C8) was only identified in the FF samples (53.57% detection frequency), while 6:2 fluorotelomer sulfonic acid (6:2 FTS) (6.12% detection frequency) and perfluoro(2-((6-chlorohexyl)oxy)ethanesulfonic acid) (9Cl-PF3ONS) (4.08% detection frequency) were found in only a few people in the DW cohort, suggesting the two cohorts had unique exposures (Fig. 3B). Interestingly, FOSA has been used for firefighting AFFFs, as well as waterproof textiles, circuit boards, and other consumer products and has previously been detected in firefighter serum samples by Dobraca *et al.*, 2015 with significantly elevated levels in individuals 50 years or older.^10,21,68–71^ Statistical analyses were then performed for each PFAS detected in at least 3 samples from both groups. The SSA comparisons showed the median peak area relative abundance of perfluoropentane sulfonic acid (PFPeS, C5) and perfluoroheptane sulfonic acid (PFHpS, C7) were statistically higher in the DW cohort, whereas perfluorohexadecanoic acid (PFHxDA, C16) was statistically higher in the FF cohort (Wilcoxon rank-sum test, Bonferroni; *p*_adj_ < 0.05) (Fig. 3C); however, differences in relative abundances between analytes do not necessarily mean differences in concentrations (*e.g.*, a lower ratio for FOSA does not necessarily mean it is present at a lower concentration than PFHpS). Unfortunately, there is very limited information available on the uses of PFHxDA and why the FF cohort would have such high exposure aside from reports of its presence in ski wax, so we hypothesize it could also be in firetruck wax, and to our knowledge, no current studies have demonstrated elevated levels of PFHxDA in firefighters.^10,72^ Additionally, the five PFAS included in the previous NHANES comparison were again statistically significant (Table S12), further supporting previous findings from the targeted quantitative analysis. These results should therefore be used to encourage the quantitation of additional PFAS in more human samples to further assess their health effects.

### 3.3 Lipid analysis

PFAS and lipids have been linked in multiple studies, including the targeted study on the DW cohorts which showed positive associations with serum PFAS levels and cholesterol.^19,44,73^ Additionally, PFOS and PFOA have demonstrated positive correlations with total triglycerides and total cholesterol levels in humans.^74–79^ Importantly, total cholesterol and non-HDL cholesterol increased with serum PFOA and PFHxS exposure for the drinking water cohort assessed in this study.^19^ However, the full extent of how PFAS impact the broader human lipidome beyond those included in traditional clinical tests, like total triglycerides and cholesterol, is not well defined. Here, we utilized multidimensional LC-IMS-CID-MS analyses and an in-house lipid library of 877 lipids to perform SSA on lipids from five categories (fatty acyls, glycerolipids, glycerophospholipids, sphingolipids, and sterols). 387 unique lipids were identified in all samples at a Schymanski confidence level of 2 or 3, and these identifications included lipids from all five categories and 15 lipid classes (Table S13).^80^ Statistical analyses were then performed to investigate potential correlations between PFAS serum levels and lipid changes.

### 3.4 Lipid changes in exposure groups

**Fig. 4:**
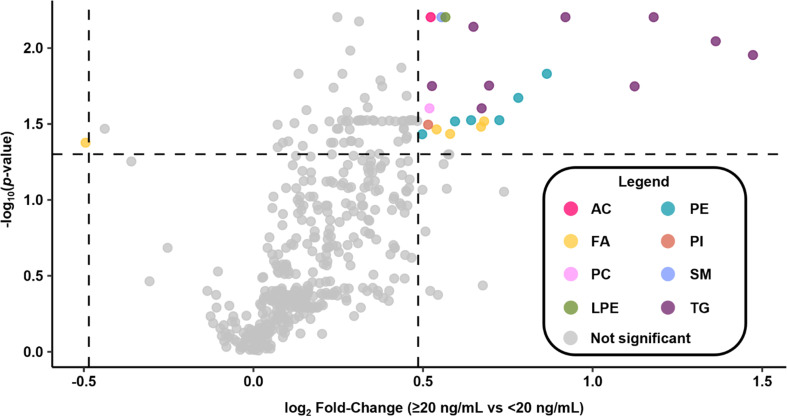
Volcano plot illustrating lipids detected and differentially expressed in the ≥20 ng mL^−1^*versus* <20 ng mL^−1^ PFAS in serum comparison. Colored dots represent statistically significant lipids by their indicated class (*p*_adj_ < 0.05 and log_2_ FC > |0.49|) and grey dots indicate species not deemed statistically significant. Of the statistically significant lipids with increased concentrations, 37.5% are triglycerides and 25.0% are phosphatidylethanolamines. The legend abbreviations correspond to lipid classes as follows: acyl carnitine (AC), fatty acyl (FA), phosphatidylcholine (PC), lysophosphatidylethanolamine (LPE), phosphatidylethanolamine (PE), phosphatidylinositol (PI), sphingomyelin (SM), and triglyceride (TG).

A linear mixed-effects model was applied to assess if the Σ7 PFAS concentrations, age, or sex are predictors of the lipid abundances while applying a random effect for the individual samples to account for non-independence between lipid abundances from the same individual. Σ7 PFAS concentrations, age, and sex were found to be statistically significant predictors (*p* < 0.05, Table S14) meaning that each of these had a significant relationship with lipid abundances when the other two are held constant (*e.g.*, PFAS sum had a significant relationship with lipid abundance when holding the effects of age and sex constant). To further explore if high PFAS serum concentrations correlated with lipid changes, the participants in this study were split based on their PFAS levels regardless of their original exposure group. According to the National Academies PFAS Exposure Guidance Report, if summed PFAS serum concentrations for 7 PFAS (Σ7 PFAS) in blood (NHANES PFAS + perfluoroundecanoic acid (PFUdA) + methylperfluoroctane sulfonamideoacetic acid (MeFOSAA)) are ≥20 ng mL^−1^, individuals should reduce PFAS exposures and talk to their physicians about screening for dyslipidemia, thyroid function, and certain cancers.^50^ The 78 participants in this study were split into exposure cohorts with 34 having Σ7 values ≥20 ng mL^−1^ and 44 with less. When comparing the Σ7 PFAS exposure cohorts, 25 lipids of the 387 identified in all samples were deemed statistically significant (Mann–Whitney *U* test, Benjamini–Hochberg; *p*_adj_ < 0.05 and log_2_ FC > |0.49|) (Table S15), and specifically 24 of the 25 increased in abundance in the ≥20 ng mL^−1^ group (Fig. 4). The statistically significant lipids cover 4 categories and 8 classes including 1 acylcarnitine (AC), 5 fatty acyls (FA), 1 phosphatidylcholine (PC), 1 lysophosphatidylethanolamine (LPE), 6 phosphatidylethanolamines (PE), 1 phosphatidylinositol (PI), 1 sphingomyelin (SM), and 9 triglycerides (TG). Of particular interest, TGs comprise 19.1% of all detected lipids but 37.5% of the lipids with increased levels due to higher PFAS serum concentrations. This is an important finding as total TGs are known to be associated with PFAS exposure and high TG levels increase the risk of heart disease, strokes, and obesity.^46,81–85^ This study was also able to identify the 9 specific TGs that correspond to this increase to further understand potential molecular mechanisms (Table S15). In this study, PEs were also found to comprise 10.3% of detected lipids but 25.0% of the lipids with increased levels. While PEs have yet to be associated with PFAS exposure in human serum, a study by Stoffels *et al.* demonstrated that PEs, along with TGs, are increased with PFOA exposure in mice liver.^73–79,86–88^ PEs are also the second most abundant glycerophospholipid in mammalian cells, following PCs, and are essential for cell membrane structure, cellular division, and hepatic secretion of very low-density lipoproteins (VLDL).^89,90^ Abnormal PC/PE ratios have been linked to the development of non-alcoholic fatty liver disease (NAFLD), liver failure, and reduced liver regeneration post-surgery, so the ≥20 ng mL^−1^ group should be carefully monitored for adverse health effects.^89,91–93^

One limitation to this study and the lipidomic analyses is that the two cohorts are not evenly distributed by sex or age and many of the highly exposed individuals were from the DW cohort. For example, 83% of the FF samples are males whereas the DW cohort is only 37% male. Furthermore, the median FF age is 35.5 years compared to the DW median age of 58 years. Because the DW cohort comprises 97% of the ≥20 ng mL^−1^ group (33 of the 34 were from the DW cohort), the majority of the ≥20 ng mL^−1^ group is both female and older in age, which may lead to altered lipid levels compared to the younger males. To account for these potential lipid differences, female-only samples from the DW cohort were split into the ≥20 *versus* <20 ng mL^−1^ exposure groups and reassessed. This resulted in 21 DW females in the ≥20 ng mL^−1^ exposure group and 10 in the <20 ng mL^−1^ group with age ranges that overlapped. Notably, the DW females in the <20 ng mL^−1^ group have primarily lived in their current homes for less than 7 years (70%) whereas the females in the ≥20 ng mL^−1^ group have primarily lived in their homes for more than 7 years (67%), therefore age and years in home with contaminated drinking water sources cannot be decoupled. A comparison of the DW female analysis to the all-sample analysis illustrated 13 of the original 25 significant lipids had greater than a 1.4-fold increase in the ≥20 ng mL^−1^ individuals for both studies (Table S16), though not statistically significant (*p*_adj_ > 0.05). Specifically, 3 PEs and 6 TGs (the classes of previous interest) were upregulated with species including: PE(16:0_22:6), PE(18:0_22:6), PE(O-18:0/22:6), TG(56:8), TG(58:11), TG(60:10), TG(60:11), TG(60:12) and TG(60:13). Since these overlapping lipids account for covariates such as occupation, sex and age, they are of great interest for future mechanistic studies into lipid changes due to PFAS exposure and how these could affect health beyond what is already known about PE and TG dysregulation. In addition to sex and age, underlying health conditions and metabolic behaviors may also vary amongst these populations and could potentially be drivers in bodily PFAS retention and excretion, as well as the observed lipidomic changes. However, specific health data was not included in the scope of this study and should be monitored in future PFAS exposure studies.

### 3.5 Species correlations

**Fig. 5:**
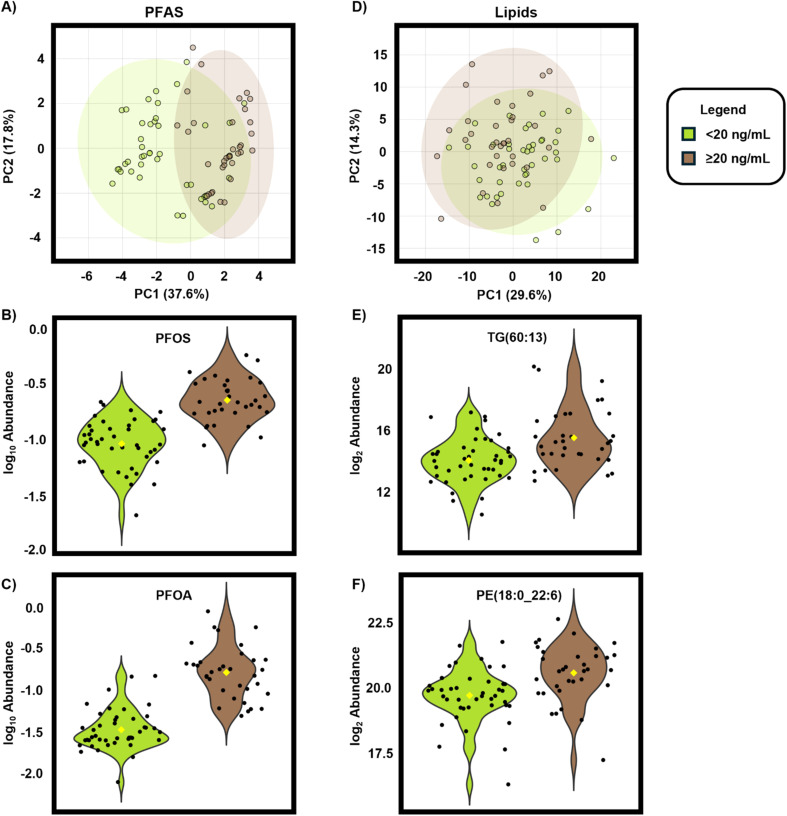
PFAS and lipid suspect screening data illustrate changes for the ≥20 ng mL^−1^ and <20 ng mL^−1^ PFAS serum groups. Principal component analysis for (A) PFAS suspect screening data and violin plots showing key PFAS, (B) PFOS and (C) PFOA, which distinguish the groups. (D) Principal component analysis for lipid suspect screening data and violin plots for key lipids including (E) TG(60:13) and (F) PE(18:0_22:6) which show elevated levels for each analyte in the ≥20 ng mL^−1^ group.

To further understand PFAS/lipid correlations, we also assessed lipid abundance relationships with individual PFAS. Here, peak abundances for all 16 SSA PFAS were imported to MetaboAnalyst 6.0 for a log_10_ transformation prior to principal component analysis (PCA) between the ≥20 ng mL^−1^ and <20 ng mL^−1^ exposure groups from all samples to determine if there was any group separation (Fig. 5A).^58^ From the PCA plots, PFOS and PFOA were determined to be key PFAS drivers which separated the groups (Fig. 5B and C). Next, the log_2_ peak abundances for all 387 SSA lipids were imported to MetaboAnalyst 6.0 to observe if lipids also separated the ≥20 ng mL^−1^ and <20 ng mL^−1^ groups (Fig. 5D). Here, TG(60:13) and PE(18:0_22:6) are shown as examples of key lipid drivers of separation (Fig. 5E and F) but there were also additional lipid drivers. For example, the PCA loadings plots reveal that of the top 20 features with the largest magnitude loadings scores for PC1, 18 were TGs (90%) along with 1 PE and 1 PC (5% each). Similarly, of the top 20 features for PC2, 9 were TGs (45%) and 4 were PEs (20%) with the remainder being 3 PCs (15%), 2 FAs (10%), and 1 cholesterol ester (CE) (5%). These results further support that TG and PE perturbations are linked to high serum PFAS levels.

**Fig. 6:**
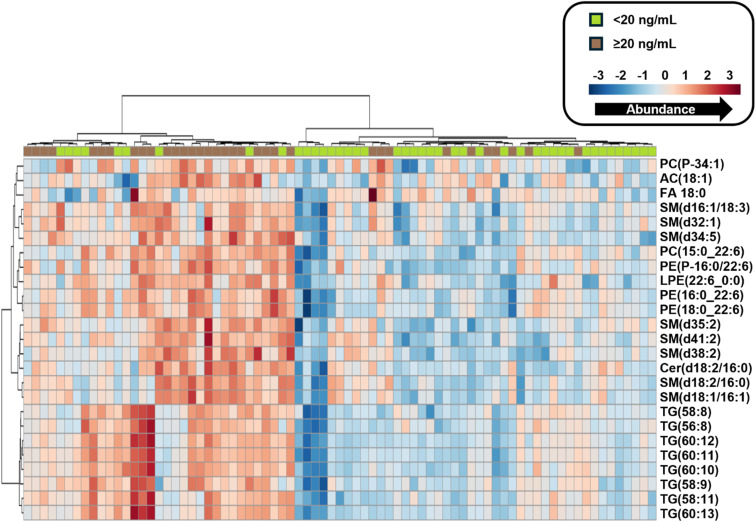
Unsupervised hierarchical clustering of the top 25 lipid features for the ≥20 ng mL^−1^*versus* <20 ng mL^−1^ PFAS serum groups. These include lipids from 7 classes, primarily triglycerides and sphingomyelins. The color gradient represents the magnitude of analyte abundance, with red signifying higher values and blue signifying lower values. The exposure groups are shown in brown (≥20 ng mL^−1^) and green (<20 ng mL^−1^). All 25 lipid abundances shown trend higher in the ≥20 ng mL^−1^ group and all 387 detected lipids were used for Spearman correlations.

Another method for determining similarity between data points, regardless of the assigned groups, is hierarchical clustering. Hierarchical clustering was applied to all lipid abundances from all samples to observe how the 20 ng mL^−1^*versus* <20 ng mL^−1^ samples differentiate.^58^ When observing the top 25 features based on hierarchical clustering (Fig. 6), the two exposure cohorts separated by 8 TGs, 8 SMs, 3 PEs, 1 LPE, 1 AC, 1 FA, 2 PCs, and 1 ceramide (Cer) which trend with higher abundances in the ≥20 ng mL^−1^ samples, although there is some intermixing with the <20 ng mL^−1^ group. Spearman correlation tests were then performed on all 387 lipid features and 8 PFAS from SSA with detection frequencies ≥50% including PFPeS, PFHxS, PFHpS, PFOS, PFHpA, PFOA, PFNA, and PFDA to determine if correlations between individual PFAS and lipid species exist. Here, 5 PFAS were found to positively correlate with lipids, with Spearman rho values (*r*_s_) between |0.4| and |0.69| (*p* < 0.05) amounting to 77 positive correlations and 2 negative. Based on the Dancey and Reidy interpretation of *r*_s_, these values suggest that there is a statistically significant and moderate linear relationship (positive or negative) between these PFAS and lipid abundances (Table S17).^94,95^ PFNA had the most positive correlations which included 37 lipids (14 SMs, 9 TGs, 5 PEs, 3 FAs, 3 LPEs, and 3 PCs). Of these, 16 lipids previously had increased abundances with the ≥20 ng mL^−1^ exposure group including all 9 TGs, 3 PEs, 1 SM, 1 FA, 1 LPE, and 1 PC. PFOA had the second highest number of positive correlations at 26 (11 SMs, 7 FAs, 3 LPEs, 2 ACs, 2 PCs, and 1 PE), followed by PFDA with 10 (6 SMs, 2 FAs, 1 LPE, and 1 PE), PFHxS with 2 FAs, and PFHpS with 1 AC and 1 FA (Table S18). PFHpA and PFOS had no positive correlations with any lipids; however, PFHpA was the only PFAS with any negative correlations, and they were with 2 FAs. Of specific note was the prevalence of PFCA correlations with sphingomyelins, as sphingomyelins modulate cellular activity like immune response, tissue development, and cell recognition and apoptosis.^96,97^ Elevated levels of sphingomyelins may also lead to increased risk for several disorders and diseases including insulin resistance, coronary heart disease, fatty liver disease, obesity, or age-related cognitive decline, making it crucial to understand the how PFAS (and specifically PFCAs) alter sphingomyelins.^98–100^

## 4 Conclusions

In this study, PFAS and lipidomic analyses were performed on serum samples collected from humans with elevated PFAS exposures due to drinking water contamination and firefighter occupational exposure. When comparing the concentrations of 5 PFAS measured from NHANES, the participants with drinking water exposure had PFAS serum concentrations greater than the national median and firefighter cohort. In contrast, the firefighters were higher than the national median for PFHxS, but lower for PFNA and PFDA. Suspect screening analyses then detected 6 additional PFAS in the participants not previously measured in the targeted studies. These included PFPeS, PFHpS, PFDS, and PFHxDA detected in both cohorts, FOSA (detected only in the firefighters) and 9Cl-PF3ONS (detected only in the drinking water group). To understand if lipid changes were associated with PFAS serum levels, the participants were regrouped into exposure cohorts in accordance with the National Academies PFAS Exposure Guidance Report (Σ7 values being either ≥20 ng mL^−1^ or <20 ng mL^−1^). Lipidomic studies revealed 24 statistically significant and increasing lipids for the participants with ≥20 ng mL^−1^ values, where triglycerides and phosphatidylethanolamines comprised 62.5% of these. Further investigation into individual PFAS species and lipid correlations also revealed that PFOA, PFNA, and PFDA (PFCAs C8-10) have 73 total positive correlations with lipids, primarily sphingomyelins and triglycerides.

This study demonstrates the necessity for further research into biological changes due not only to total PFAS levels, but to individual PFAS classes and species, as the biological responses may vary based on exposure profiles. Moreover, this study contributes to the ongoing efforts to characterize the impacts of PFAS on human health and metabolic pathways. It also reveals that total PFAS serum levels are linked to increased lipid abundances, primarily TGs and PEs, whereas elevated PFCA levels are associated with higher levels of TGs and SMs. These findings should be used to inform future clinical and toxicological studies of specific PFAS of interest for health and disease monitoring. Applying both PFAS and lipidomic analyses, in addition to other omics, to future studies will also reveal more information regarding altered biological mechanisms due to PFAS exposure. While this study allowed for the assessment of biological responses due to two distinct PFAS exposure pathways in humans, these individuals likely had otherwise overlapping lifestyles such as food and resource availabilities due to their close geographical proximities. Therefore, further research is encouraged to assess individuals from other geographical regions with different exposure pathways, environments, and lifestyles.

## Ethical statement

The Duke University Institutional Review Board reviewed and approved all study protocols prior to recruitment, and all study participants provided informed consent.

## Author contributions

Ashlee T. Falls: conceptualization, investigation, writing – original draft, visualization, software, formal analysis. Anna K. Boatman: conceptualization, methodology, validation. Jack P. Ryan: conceptualization, validation. Amie M. Solosky: conceptualization, methodology, validation. James N. Dodds: conceptualization, methodology. Jessie R. Chappel: software, validation, formal analysis. Allison N. Fry: software, validation, formal analysis. Kaylie I. Kirkwood-Donelson: methodology. Heather M. Stapleton: resources, conceptualization, investigation, writing – review & editing. Erin S. Baker: supervision, project administration, funding acquisition, conceptualization, resources, writing – review & editing.

## Conflicts of interest

The authors declare no competing financial interest.

## Supplementary Material

VA-005-D5VA00483G-s001

VA-005-D5VA00483G-s002

VA-005-D5VA00483G-s003

## Data Availability

All data are available in the main text or the supplementary information (SI). Skyline files containing LC-IMS-HRMS data are available at (https://panoramaweb.org/pfaslipidshumans.url) and raw data files are available at (https://doi.org/10.25345/C5TT4G63P). Supplementary information: the data includes consumables and part numbers, LC-IMS-MS methods and settings, and additional experimental and statistical details (XLSX). Detailed extraction and instrumentation methods are also provided including a figure of the percent composition of detected lipids by class (PDF). Additional annotation details are available in the Lipid Reporting Checklist included (PDF). See DOI: https://doi.org/10.1039/d5va00483g.

## References

[cit1] Buck R. C., Franklin J., Berger U., Conder J. M., Cousins I. T., De Voogt P., Jensen A. A., Kannan K., Mabury S. A., Van Leeuwen S. P. (2011). Perfluoroalkyl and Polyfluoroalkyl Substances in the Environment: Terminology, Classification, and Origins. Integr. Environ. Assess. Manage..

[cit2] Wang Z., Buser A. M., Cousins I. T., Demattio S., Drost W., Johansson O., Ohno K., Patlewicz G., Richard A. M., Walker G. W. (2021). *et al.*, A New OECD Definition for Per- and Polyfluoroalkyl Substances. Environ. Sci. Technol..

[cit3] OECD , Reconciling Terminology of the Universe of Per- and Polyfluoroalkyl Substances: Recommendations and Practical Guidance, OECD Series on Risk Management of Chemicals, OECD Publishing, 2021

[cit4] Schymanski E. L., Zhang J., Thiessen P. A., Chirsir P., Kondic T., Bolton E. E. (2023). Per- and Polyfluoroalkyl Substances (PFAS) in PubChem: 7 Million and Growing. Environ. Sci. Technol..

[cit5] NCBI/NLM/NIH , PubChem Website, https://pubchem.ncbi.nlm.nih.gov/(accessed 2025-01-13)

[cit6] Fenton S. E., Ducatman A., Boobis A., DeWitt J. C., Lau C., Ng C., Smith J. S., Roberts S. M. (2021). Per- and Polyfluoroalkyl Substance Toxicity and Human Health Review: Current State of Knowledge and Strategies for Informing Future Research. Environ. Toxicol. Chem..

[cit7] Herzke D., Olsson E., Posner S. (2012). Perfluoroalkyl and polyfluoroalkyl substances (PFASs) in consumer products in Norway - a pilot study. Chemosphere.

[cit8] Whitehead H. D., Venier M., Wu Y., Eastman E., Urbanik S., Diamond M. L., Shalin A., Schwartz-Narbonne H., Bruton T. A., Blum A. (2021). *et al.*, Fluorinated compounds in North American cosmetics. Environ. Sci. Technol. Lett..

[cit9] Kotthoff M., Müller J., Jürling H., Schlummer M., Fiedler D. (2015). Perfluoroalkyl and polyfluoroalkyl substances in consumer products. Environ. Sci. Pollut. Res. Int..

[cit10] Glüge J., Scheringer M., Cousins I. T., DeWitt J. C., Goldenman G., Herzke D., Lohmann R., Ng C. A., Trier X., Wang Z. (2020). An overview of the uses of per- and polyfluoroalkyl substances (PFAS). Environ. Sci.: Processes Impacts.

[cit11] Ramírez Carnero A., Lestido-Cardama A., Vazquez Loureiro P., Barbosa-Pereira L., Rodríguez Bernaldo de Quirós A., Sendón R. (2021). Presence of Perfluoroalkyl and Polyfluoroalkyl Substances (PFAS) in Food Contact Materials (FCM) and Its Migration to Food. Foods.

[cit12] Høisæter Å., Pfaff A., Breedveld G. D. (2019). Leaching and transport of PFAS from aqueous film-forming foam (AFFF) in the unsaturated soil at a firefighting training facility under cold climatic conditions. J. Contam. Hydrol..

[cit13] Hu X. C., Andrews D. Q., Lindstrom A. B., Bruton T. A., Schaider L. A., Grandjean P., Lohmann R., Carignan C. C., Blum A., Balan S. A. (2016). *et al.*, Detection of Poly- and Perfluoroalkyl Substances (PFASs) in U.S. Drinking Water Linked to Industrial Sites, Military Fire Training Areas, and Wastewater Treatment Plants. Environ. Sci. Technol. Lett..

[cit14] Moody C. A., Field J. A. (2000). Perfluorinated Surfactants and the Environmental Implications of Their Use in Fire-Fighting Foams. Environ. Sci. Technol..

[cit15] Houtz E. F., Higgins C. P., Field J. A., Sedlak D. L. (2013). Persistence of Perfluoroalkyl Acid Precursors in AFFF-Impacted Groundwater and Soil. Environ. Sci. Technol..

[cit16] Mcgarr J. T., Mbonimpa E. G., Mcavoy D. C., Soltanian M. R. (2023). Fate and Transport of Per- and Polyfluoroalkyl Substances (PFAS) at Aqueous Film Forming Foam (AFFF) Discharge Sites: A Review. Soil Syst..

[cit17] Coffin E. S., Reeves D. M., Cassidy D. P. (2023). PFAS in municipal solid waste landfills: Sources, leachate composition, chemical transformations, and future challenges. Curr. Opin. Environ. Sci. Health.

[cit18] Liu Y., Robey N. M., Bowden J. A., Tolaymat T. M., da Silva B. F., Solo-Gabriele H. M., Townsend T. G. (2021). From Waste Collection Vehicles to Landfills: Indication of Per- and Polyfluoroalkyl Substance (PFAS) Transformation. Environ. Sci. Technol. Lett..

[cit19] Hall S. M., Zhang S., Tait G. H., Hoffman K., Collier D. N., Hoppin J. A., Stapleton H. M. (2023). PFAS levels in paired drinking water and serum samples collected from an exposed community in Central North Carolina. Sci. Total Environ..

[cit20] Hoxie T. E. S. , Assessing Exposure to Per- and Polyfluoroalkyl Substances in the Indoor and Ambient Environment Utilizing Silicone Wristbands, 2024, https://hdl.handle.net/10161/32628

[cit21] Mazumder N., Hossain M. T., Jahura F. T., Girase A., Hall A. S., Lu J., Ormond R. B. (2023). Firefighters' exposure to per-and polyfluoroalkyl substances (PFAS) as an occupational hazard: A review. Front. Mater..

[cit22] U.S. EPA , Final PFAS National Primary Drinking Water Regulation, 2024

[cit23] U.S. EPA , Per- and Polyfluoroalkyl Substances (PFAS) NPDWR Implementation, 2025, https://www.epa.gov/dwreginfo/pfas-rule-implementation (accessed 2025 November 5)

[cit24] U.S. EPA , EPA Announces It Will Keep Maximum Contaminant Levels for PFOA, PFOS, 2025, https://www.epa.gov/newsreleases/epa-announces-it-will-keep-maximum-contaminant-levels-pfoa-pfos (accessed 2025 November 5)

[cit25] U.S. EPA , CompTox Chemicals Dashboard, CompTox Chemicals Dashboard, 2022

[cit26] Fear, C. C. and Clinic, U. C. B. E. L, Complaint Filed with United Nations about PFAS Contamination of Drinking Water, 2023

[cit27] Khalil N., Ducatman A. M., Sinari S., Billheimer D., Hu C., Littau S., Burgess J. L. (2020). Per- and Polyfluoroalkyl Substance and Cardio Metabolic Markers in Firefighters. J. Occup. Environ. Med..

[cit28] Mitchell C. L., Hollister J., Fisher J. M., Beitel S. C., Ramadan F., O'Leary S., Fan Z. T., Lutrick K., Burgess J. L., Ellingson K. D. (2025). Differences in serum concentrations of per-and polyfluoroalkyl substances by occupation among firefighters, other first responders, healthcare workers, and other essential workers in Arizona, 2020–2023. J. Expo. Sci. Environ. Epidemiol..

[cit29] Graber J. M., Black T. M., Shah N. N., Caban-Martinez A. J., Lu S.-E., Brancard T., Yu C. H., Turyk M. E., Black K., Steinberg M. B. (2021). *et al.*, Prevalence and Predictors of Per- and Polyfluoroalkyl Substances (PFAS) Serum Levels among Members of a Suburban US Volunteer Fire Department. Int. J. Environ. Res. Publ. Health.

[cit30] Nilsson S., Smurthwaite K., Aylward L. L., Kay M., Toms L. M., King L., Marrington S., Barnes C., Kirk M. D., Mueller J. F. (2022). *et al.*, Serum concentration trends and apparent half-lives of per- and polyfluoroalkyl substances (PFAS) in Australian firefighters. Int. J. Hyg Environ. Health.

[cit31] Jin C., Sun Y., Islam A., Qian Y., Ducatman A. (2011). Perfluoroalkyl Acids Including Perfluorooctane Sulfonate and Perfluorohexane Sulfonate in Firefighters. J. Occup. Environ. Med..

[cit32] Burgess J. L., Fisher J. M., Nematollahi A., Jung A. M., Calkins M. M., Graber J. M., Grant C. C., Beitel S. C., Littau S. R., Gulotta J. J. (2023). *et al.*, Serum per- and polyfluoroalkyl substance concentrations in four municipal US fire departments. Am. J. Ind. Med..

[cit33] Holmquist H., Schellenberger S., van der Veen I., Peters G. M., Leonards P. E., Cousins I. T. (2016). Properties, performance and associated hazards of state-of-the-art durable water repellent (DWR) chemistry for textile finishing. Environ. Int..

[cit34] Peaslee G. F., Wilkinson J. T., McGuinness S. R., Tighe M., Caterisano N., Lee S., Gonzales A., Roddy M., Mills S., Mitchell K. (2020). Another Pathway for Firefighter Exposure to Per- and Polyfluoroalkyl Substances: Firefighter Textiles. Environ. Sci. Technol. Lett..

[cit35] Demers P. A., DeMarini D. M., Fent K. W., Glass D. C., Hansen J., Adetona O., Andersen M. H., Freeman L. E. B., Caban-Martinez A. J., Daniels R. D. (2022). *et al.*, Carcinogenicity of occupational exposure as a firefighter. Lancet Oncol..

[cit36] IARC Working Group on the Evaluation of Carcinogenic Risks to Humans , Painting, Firefighting, and Shiftwork, International Agency for Research on Cancer, Lyon, France, 2010, IARC Monographs on the Evaluation of Carcinogenic Risks to Humans, No. 98, https://www.ncbi.nlm.nih.gov/books/NBK326814/PMC4781497 21381544

[cit37] IARC Working Group on the Evaluation of Carcinogenic Risks to Humans , IARC Monographs on the Evaluation of Carcinogenic Risks to Humans, in Benzene, International Agency for Research on Cancer, 2018, For more information contact publications@iarc.fr

[cit38] IARC Working Group on the Evaluation of Carcinogenic Risks to Humans , Overall Evaluations of Carcinogenicity: An Updating of IARC Monographs Volumes 1 to 42, International Agency for Research on Cancer, Lyon, France, 1987, (IARC MONOGRAPHS ON THE EVALUATION OF CARCINOGENIC RISKS TO HUMANS, No. Supplement 7), available from: https://www.ncbi.nlm.nih.gov/books/NBK533509/3482203

[cit39] IARC Working Group on the Evaluation of Carcinogenic Risks to Humans , IARC Monographs on the Identification of Carcinogenic Hazards to Humans, in Perfluorooctanoic Acid (PFOA) and Perfluorooctanesulfonic Acid (PFOS), International Agency for Research on Cancer, 2025, For more information contact publications@iarc.who.int

[cit40] IARC Working Group on the Evaluation of Carcinogenic Risks to Humans , IARC Monographs on the Identification of Carcinogenic Hazards to Humans, in Occupational Exposure as a Firefighter, International Agency for Research on Cancer, 2023, For more information contact publications@iarc.fr

[cit41] Barry V., Winquist A., Steenland K. (2013). Perfluorooctanoic Acid (PFOA) Exposures and Incident Cancers among Adults Living Near a Chemical Plant. Environ. Health Perspect..

[cit42] Chang E. T., Adami H. O., Boffetta P., Wedner H. J., Mandel J. S. (2016). A critical review of perfluorooctanoate and perfluorooctanesulfonate exposure and immunological health conditions in humans. Crit. Rev. Toxicol..

[cit43] Roth K., Yang Z., Agarwal M., Liu W., Peng Z., Long Z., Birbeck J., Westrick J., Liu W., Petriello M. C. (2021). Exposure to a mixture of legacy, alternative, and replacement per- and polyfluoroalkyl substances (PFAS) results in sex-dependent modulation of cholesterol metabolism and liver injury. Environ. Int..

[cit44] Kirkwood-Donelson K. I., Chappel J., Tobin E., Dodds J. N., Reif D. M., DeWitt J. C., Baker E. S. (2024). Investigating mouse hepatic lipidome dysregulation following exposure to emerging per- and polyfluoroalkyl substances (PFAS). Chemosphere.

[cit45] Conroy M. J., Andrews R. M., Andrews S., Cockayne L., Dennis E. A., Fahy E., Gaud C., Griffiths W. J., Jukes G., Kolchin M., Mendivelso K., Lopez-Clavijo A. F., Ready C., Subramaniam S., O'Donnell V. B. (2024). LIPID MAPS: update to databases and tools for the lipidomics community. Nucleic Acids Res..

[cit46] Dunder L., Lind P. M., Salihovic S., Stubleski J., Kärrman A., Lind L. (2022). Changes in plasma levels of per- and polyfluoroalkyl substances (PFAS) are associated with changes in plasma lipids - A longitudinal study over 10 years. Environ. Res..

[cit47] Hoxie T., Zhang S., Cooper E. M., Ehrlich D., Herkert N., Hoffman K., Stapleton H. M. (2025). Assessing Nondietary Exposure to Per- and Polyfluoroalkyl Substances (PFASs) in Firefighters Using Silicone Wristbands. Environ. Health.

[cit48] Folch J., Lees M., Sloane Stanley G. H. (1957). A simple method for the isolation and purification of total lipides from animal tissues. J. Biol. Chem..

[cit49] ATSDR , Final Report: Findings across Ten Exposure Assessment (EA) Sites, Agency for Toxic Substances and Disease Registry, 2022

[cit50] National Academies of Sciences Engineering and Medicine (U.S.) , Committee on the Guidance on PFAS Testing and Health Outcomes; National Academies of Sciences Engineering and Medicine (U.S.). Board on Population Health and Public Health Practice; National Academies of Sciences Engineering and Medicine (U.S.). Board on Environmental Studies and Toxicology, Guidance on PFAS Exposure, Testing, and Clinical Follow-Up, National Academies Press, 2022

[cit51] Boatman A. K., Chappel J. R., Polera M. E., Dodds J. N., Belcher S. M., Baker E. S. (2024). Assessing Per- and Polyfluoroalkyl Substances in Fish Fillet Using Non-Targeted Analyses. Environ. Sci. Technol..

[cit52] Phillips A. L., Peter K. T., Sobus J. R., Fisher C. M., Manzano C. A., McEachran A. D., Williams A. J., Knolhoff A. M., Ulrich E. M. (2023). Standardizing non-targeted analysis reporting to advance exposure science and environmental epidemiology. J. Expo. Sci. Environ. Epidemiol..

[cit53] Foster M., Rainey M., Watson C., Dodds J. N., Kirkwood K. I., Fernández F. M., Baker E. S. (2022). Uncovering PFAS and Other Xenobiotics in the Dark Metabolome Using Ion Mobility Spectrometry, Mass Defect Analysis, and Machine Learning. Environ. Sci. Technol..

[cit54] Boatman A. K. , Chappel J. R., Kirkwood-Donelson K. I., Fleming J. F., Reif D. M., Schymanski E. L., Rager J. E. and Baker E. S., Updated Guidance for Communicating PFAS Identification Confidence with Ion Mobility Spectrometry, bioRxiv, 2025, preprint, 10.1101/2025.01.27.634925 PMC12424050 40808630

[cit55] Marvel S., To K., Grimm F., Wright F., Rusyn I., Reif D. (2018). ToxPi Graphical User Interface 2.0: Dynamic exploration, visualization, and sharing of integrated data models. BMC Bioinf..

[cit56] Reif D., Martin M., Tan S., Houck K., Judson R., Richard A., Knudsen T., Dix D., Kavlock R. (2010). Endocrine profiling and prioritization of environmental chemicals using ToxCast data. Environ. Health Perspect..

[cit57] Kirkwood K. I., Pratt B. S., Shulman N., Tamura K., MacCoss M. J., MacLean B. X., Baker E. S. (2022). Utilizing Skyline to analyze lipidomics data containing liquid chromatography, ion mobility spectrometry and mass spectrometry dimensions. Nat. Protoc..

[cit58] Xia J., Psychogios N., Young N., Wishart D. S. (2009). MetaboAnalyst: a web server for metabolomic data analysis and interpretation. Nucleic Acids Res..

[cit59] Khan E. A., Grønnestad R., Krøkje Å., Bartosov Z., Johanson S. M., Müller M. H. B., Arukwe A. (2023). Alteration of hepato-lipidomic homeostasis in A/J mice fed an environmentally relevant PFAS mixture. Environ. Int..

[cit60] Pétré M. A., Salk K. R., Stapleton H. M., Ferguson P. L., Tait G., Obenour D. R., Knappe D. R. U., Genereux D. P. (2022). Per- and polyfluoroalkyl substances (PFAS) in river discharge: Modeling loads upstream and downstream of a PFAS manufacturing plant in the Cape Fear watershed, North Carolina. Sci. Total Environ..

[cit61] National Heart, Lung, and Blood Institute , High Blood Triglycerides, U.S. Department of Health and Human Services, National Institutes of Health, 2025, https://www.durhamnc.gov/5312/PFAS-Drinking-Water-Standards

[cit62] Weed R. A., Campbell G., Brown L., May K., Sargent D., Sutton E., Burdette K., Rider W., Baker E. S., Enders J. R. (2024). Non-Targeted PFAS Suspect Screening and Quantification of Drinking Water Samples Collected through Community Engaged Research in North Carolina's Cape Fear River Basin. Toxics.

[cit63] Nakayama S., Strynar M. J., Helfant L., Egeghy P., Ye X., Lindstrom A. B. (2007). Perfluorinated Compounds in the Cape Fear Drainage Basin in North Carolina. Environ. Sci. Technol..

[cit64] Herkert N. J., Merrill J., Peters C., Bollinger D., Zhang S., Hoffman K., Ferguson P. L., Knappe D. R. U., Stapleton H. M. (2020). Assessing the Effectiveness of Point-of-Use Residential Drinking Water Filters for Perfluoroalkyl Substances (PFASs). Environ. Sci. Technol. Lett..

[cit65] Hopkins Z. R., Sun M., Dewitt J. C., Knappe D. R. U. (2018). Recently Detected Drinking Water Contaminants: GenX and Other Per- and Polyfluoroalkyl Ether Acids. J. AWWA.

[cit66] Pétré M.-A., Genereux D. P., Koropeckyj-Cox L., Knappe D. R. U., Duboscq S., Gilmore T. E., Hopkins Z. R. (2021). Per- and Polyfluoroalkyl Substance (PFAS) Transport from Groundwater to Streams near a PFAS Manufacturing Facility in North Carolina, USA. Environ. Sci. Technol..

[cit67] Sun M., Arevalo E., Strynar M., Lindstrom A., Richardson M., Kearns B., Pickett A., Smith C., Knappe D. R. U. (2016). Legacy and Emerging Perfluoroalkyl Substances Are Important Drinking Water Contaminants in the Cape Fear River Watershed of North Carolina. Environ. Sci. Technol. Lett..

[cit68] National Center for Biotechnology Information , PubChem Compound Summary for CID 69785, Perfluorooctanesulfonamide, 2025

[cit69] Herzke D. , Posner S. and Olsson E., Survey, Screening and Analysis of PFC in Consumer Products, 2009

[cit70] Schulze P.-E. and Norin H., Fluorinated pollutants in all-weather clothing, Friends of the Earth Norway, 2006

[cit71] Dobraca D., Israel L., McNeel S., Voss R., Wang M., Gajek R., Park J.-S., Harwani S., Barley F., She J. (2015). *et al.*, Biomonitoring in California Firefighters:
Metals and Perfluorinated Chemicals. J. Occup. Environ. Med..

[cit72] National Center for Biotechnology Information , PubChem Compound Summar for CID 106027, Perfluorohexadecanoic Acid, 2025

[cit73] Dunder L., Salihovic S., Lind P. M., Elmståhl S., Lind L. (2023). Plasma levels of per- and polyfluoroalkyl substances (PFAS) are associated with altered levels of proteins previously linked to inflammation, metabolism and cardiovascular disease. Environ. Int..

[cit74] Steenland K., Fletcher T., Stein C. R., Bartell S. M., Darrow L., Lopez-Espinosa M.-J., Barry Ryan P., Savitz D. A. (2020). Review: Evolution of evidence on PFOA and health following the assessments of the C8 Science Panel. Environ. Int..

[cit75] Steenland K., Tinker S., Frisbee S., Ducatman A., Vaccarino V. (2009). Association of Perfluorooctanoic Acid and Perfluorooctane Sulfonate With Serum Lipids Among Adults Living Near a Chemical Plant. Am. J. Epidemiol..

[cit76] Olsen G. W., Burris J. M., Burlew M. M., Mandel J. H. (2003). Epidemiologic Assessment of Worker Serum Perfluorooctanesulfonate (PFOS) and Perfluorooctanoate (PFOA) Concentrations and Medical Surveillance Examinations. J. Occup. Environ. Med..

[cit77] Costa G., Sartori S., Consonni D. (2009). Thirty Years of Medical Surveillance in Perfluooctanoic Acid Production Workers. J. Occup. Environ. Med..

[cit78] Yu C. H., Riker C. D., Lu S.-e., Fan Z. (2020). Biomonitoring of emerging contaminants, perfluoroalkyl and polyfluoroalkyl substances (PFAS), in New Jersey adults in 2016–2018. Int. J. Hyg Environ. Health.

[cit79] Lin P.-I. D., Cardenas A., Hauser R., Gold D. R., Kleinman K. P., Hivert M.-F., Fleisch A. F., Calafat A. M., Webster T. F., Horton E. S. (2019). *et al.*, Per- and polyfluoroalkyl substances and blood lipid levels in pre-diabetic adults—longitudinal analysis of the diabetes prevention program outcomes study. Environ. Int..

[cit80] Schymanski E. L., Jeon J., Gulde R., Fenner K., Ruff M., Singer H. P., Hollender J. (2014). Identifying Small Molecules *via* High Resolution Mass Spectrometry: Communicating Confidence. Environ. Sci. Technol..

[cit81] Zeng X.-W., Qian Z., Emo B., Vaughn M., Bao J., Qin X.-D., Zhu Y., Li J., Lee Y. L., Dong G.-H. (2015). Association of polyfluoroalkyl chemical exposure with serum lipids in children. Sci. Total Environ..

[cit82] Koshy T. T., Attina T. M., Ghassabian A., Gilbert J., Burdine L. K., Marmor M., Honda M., Chu D. B., Han X., Shao Y. (2017). *et al.*, Serum perfluoroalkyl substances and cardiometabolic consequences in adolescents exposed to the World Trade Center disaster and a matched comparison group. Environ. Int..

[cit83] Miller M., Stone N. J., Ballantyne C., Bittner V., Criqui M. H., Ginsberg H. N., Goldberg A. C., Howard W. J., Jacobson M. S., Kris-Etherton P. M. (2011). *et al.*, Triglycerides and Cardiovascular Disease. Circulation.

[cit84] Akhtar N., Singh R., Kamran S., Joseph S., Morgan D., Uy R. T., Treit S., Shuaib A. (2024). Association between serum triglycerides and stroke type, severity, and prognosis. Analysis in 6558 patients. BMC Neurol..

[cit85] Carpentier A. C. (2015). Hypertriglyceridemia Associated With Abdominal Obesity. Arterioscler., Thromb., Vasc. Biol..

[cit86] Schillemans T., Bergdahl I. A., Hanhineva K., Shi L., Donat-Vargas C., Koponen J., Kiviranta H., Landberg R., Åkesson A., Brunius C. (2023). Associations of PFAS-related plasma metabolites with cholesterol and triglyceride concentrations. Environ. Res..

[cit87] National Heart, Lung, and Blood Institute , High Blood Triglycerides, U.S. Department of Health and Human Services, National Institutes of Health, p. 2023

[cit88] Stoffels C. B. A., Angerer T. B., Robert H., Poupin N., Lakhal L., Frache G., Mercier-Bonin M., Audinot J.-N. (2023). Lipidomic Profiling of PFOA-Exposed Mouse Liver by Multi-Modal Mass Spectrometry Analysis. Anal. Chem..

[cit89] van der Veen J. N., Kennelly J. P., Wan S., Vance J. E., Vance D. E., Jacobs R. L. (2017). The critical role of phosphatidylcholine and phosphatidylethanolamine metabolism in health and disease. Biochim. Biophys. Acta, Biomembr..

[cit90] Farine L., Niemann M., Schneider A., Bütikofer P. (2015). Phosphatidylethanolamine and phosphatidylcholine biosynthesis by the Kennedy pathway occurs at different sites in Trypanosoma brucei. Sci. Rep..

[cit91] Ling J., Chaba T., Zhu L.-F., Jacobs R. L., Vance D. E. (2012). Hepatic ratio of phosphatidylcholine to phosphatidylethanolamine predicts survival after partial hepatectomy in mice. Hepatology.

[cit92] Li Z., Agellon L. B., Allen T. M., Umeda M., Jewell L., Mason A., Vance D. E. (2006). The ratio of phosphatidylcholine to phosphatidylethanolamine influences membrane integrity and steatohepatitis. Cell Metab..

[cit93] Walkey C. J., Yu L., Agellon L. B., Vance D. E. (1998). Biochemical and Evolutionary Significance of Phospholipid Methylation. J. Biol. Chem..

[cit94] Akoglu H. (2018). User's guide to correlation coefficients. Turk. J. Emerg. Med..

[cit95] Dancey C. P. and Reidy J., Statistics without Maths for Psychology, Pearson education, 2007

[cit96] Dasgupta S., Ray S. K. (2017). Diverse Biological Functions of Sphingolipids in the CNS: Ceramide and Sphingosine Regulate Myelination in Developing Brain but Stimulate Demyelination during Pathogenesis of Multiple Sclerosis. J. Neurol. Psychol..

[cit97] Lee M., Lee S. Y., Bae Y.-S. (2023). Functional roles of sphingolipids in immunity and their implication in disease. Exp. Mol. Med..

[cit98] Chakraborty M. and Jiang X.-C., Sphingomyelin and Its Role in Cellular Signaling, in Advances in Experimental Medicine and Biology, Springer, Netherlands, 2013, pp 1–14 10.1007/978-94-007-6331-9_1 23775687

[cit99] Kumar M., Aguiar M., Jessel A., Thurberg B. L., Underhill L., Wong H., George K., Davidson V., Schuchman E. H. (2024). The impact of sphingomyelin on the pathophysiology and treatment response to olipudase alfa in acid sphingomyelinase deficiency. Genet. Med. Open.

[cit100] Pongrac Barlovic D., Harjutsalo V., Sandholm N., Forsblom C., Groop P.-H. (2020). Sphingomyelin and progression of renal and coronary heart disease
in individuals with type 1 diabetes. Diabetologia.

