# Supplementary material for: Increasing PFAS concentrations in human serum correlate with elevated blood lipid levels
Source: Env Sci Adv. 2026 Feb 16;5(3):885–99. doi: 10.1039/d5va00483g (PMC12921673; doi:10.1039/d5va00483g)
Supplement: VA-005-D5VA00483G-s001 [file VA-005-D5VA00483G-s001.pdf]

Supplementary Information (SI) for Environmental Science: Advances.
This journal is © The Royal Society of Chemistry 2026

Contents of Report

Created by https://lipidomicstandards.org, version v2.5.0

Separation Workflow 2
Overall study design 2
Lipid extraction 2
Analytical platform 3
Quality control 3
Method qualification and validation 3
Reporting 3

Sample Descriptions 3
Human Serum PFAS/Lipids / Human / Serum 3

Lipid Class Descriptions 4
1) Cer[M-H]- / Lipid identification 4
1) Cer[M-H]- / Lipid quantification 4
2) Cer[M+HCOO]- / Lipid identification 4
2) Cer[M+HCOO]- / Lipid quantification 5
3) Cer[M+CH3COO]- / Lipid identification 5
3) Cer[M+CH3COO]- / Lipid quantification 5
4) CL[M-H]- / Lipid identification 6
4) CL[M-H]- / Lipid quantification 6
5) CL[M-2H]2- / Lipid identification 6
5) CL[M-2H]2- / Lipid quantification 6
6) FA[M-H]- / Lipid identification 7
6) FA[M-H]- / Lipid quantification 7
7) FAHFA[M-H]- / Lipid identification 7
7) FAHFA[M-H]- / Lipid quantification 7
8) GM3[M-H]- / Lipid identification 8
8) GM3[M-H]- / Lipid quantification 8
9) GD1[M-2H]2- / Lipid identification 8
9) GD1[M-2H]2- / Lipid quantification 9
10) GM1[M-2H]2- / Lipid identification 9
10) GM1[M-2H]2- / Lipid quantification 9
11) HexCer[M-H]- / Lipid identification 9
11) HexCer[M-H]- / Lipid quantification 10
12) HexCer[M+HCOO]- / Lipid identification 10
12) HexCer[M+HCOO]- / Lipid quantification 10
13) HexCer[M+CH3COO]- / Lipid identification 11
13) HexCer[M+CH3COO]- / Lipid quantification 11
14) LPC[M+CH3COO]- / Lipid identification 11
14) LPC[M+CH3COO]- / Lipid quantification 12
15) LPE[M-H]- / Lipid identification 12
15) LPE[M-H]- / Lipid quantification 12
16) LPE O[M-H]- / Lipid identification 13
16) LPE O[M-H]- / Lipid quantification 13
17) LPG[M-H]- / Lipid identification 13
17) LPG[M-H]- / Lipid quantification 14
18) LPI[M-H]- / Lipid identification 14
18) LPI[M-H]- / Lipid quantification 14
19) PA[M-H]- / Lipid identification 15
19) PA[M-H]- / Lipid quantification 15
20) PC[M+CH3COO]- / Lipid identification 15
20) PC[M+CH3COO]- / Lipid quantification 16
21) PC O[M+CH3COO]- / Lipid identification 16
21) PC O[M+CH3COO]- / Lipid quantification 17
22) PC P[M+CH3COO]- / Lipid identification 17
22) PC P[M+CH3COO]- / Lipid quantification 17
23) PE[M-H]- / Lipid identification 17
23) PE[M-H]- / Lipid quantification 18
24) PE O[M-H]- / Lipid identification 18
24) PE O[M-H]- / Lipid quantification 19
25) PE P[M-H]- / Lipid identification 19
25) PE P[M-H]- / Lipid quantification 19
26) PG[M-H]- / Lipid identification 20
26) PG[M-H]- / Lipid quantification 20
27) PI[M-H]- / Lipid identification 20
27) PI[M-H]- / Lipid quantification 21
28) PS[M-H]- / Lipid identification 21
28) PS[M-H]- / Lipid quantification 22

29) SM[M+HCOO]- / Lipid identification . . . 22
29) SM[M+HCOO]- / Lipid quantification . . . 22
30) SM[M+CH3COO]- / Lipid identification . . . 22
30) SM[M+CH3COO]- / Lipid quantification . . . 23
31) CAR[M+H]+ / Lipid identification . . . 23
31) CAR[M+H]+ / Lipid quantification . . . 23
32) NAE[M+H]+ / Lipid identification . . . 24
32) NAE[M+H]+ / Lipid quantification . . . 24
33) CE[M+NH4]+ / Lipid identification . . . 24
33) CE[M+NH4]+ / Lipid quantification . . . 24
34) Cer[M+H]+ / Lipid identification . . . 25
34) Cer[M+H]+ / Lipid quantification . . . 25
35) DG[M+NH4]+ / Lipid identification . . . 25
35) DG[M+NH4]+ / Lipid quantification . . . 26
36) LPC[M+H]+ / Lipid identification . . . 26
36) LPC[M+H]+ / Lipid quantification . . . 26
37) LPC[M+Na]+ / Lipid identification . . . 26
37) LPC[M+Na]+ / Lipid quantification . . . 27
38) LPE[M+H]+ / Lipid identification . . . 27
38) LPE[M+H]+ / Lipid quantification . . . 28
39) LPE[M+Na]+ / Lipid identification . . . 28
39) LPE[M+Na]+ / Lipid quantification . . . 28
40) PC[M+H]+ / Lipid identification . . . 28
40) PC[M+H]+ / Lipid quantification . . . 29
41) PC[M+Na]+ / Lipid identification . . . 29
41) PC[M+Na]+ / Lipid quantification . . . 30
42) PC O[M+H]+ / Lipid identification . . . 30
42) PC O[M+H]+ / Lipid quantification . . . 30
43) PC O[M+Na]+ / Lipid identification . . . 30
43) PC O[M+Na]+ / Lipid quantification . . . 31
44) PC P[M+H]+ / Lipid identification . . . 31
44) PC P[M+H]+ / Lipid quantification . . . 31
45) PC P[M+Na]+ / Lipid identification . . . 32
45) PC P[M+Na]+ / Lipid quantification . . . 32
46) PE[M+H]+ / Lipid identification . . . 32
46) PE[M+H]+ / Lipid quantification . . . 33
47) PE[M+Na]+ / Lipid identification . . . 33
47) PE[M+Na]+ / Lipid quantification . . . 33
48) SM[M+H]+ / Lipid identification . . . 34
48) SM[M+H]+ / Lipid quantification . . . 34
49) TG[M+NH4]+ / Lipid identification . . . 34
49) TG[M+NH4]+ / Lipid quantification . . . 35

Separation Workflow

Overall study design

Title of the study	Increasing PFAS Concentrations in Human Serum Correlate with Elevated Blood Lipid Levels		
Document creation date	12/18/2025	Principal investigator	Erin Baker
Institution	University of North Carolina at Chapel Hill	Corresponding Email	erinmsb@unc.edu
Is the workflow targeted or untargeted?	Untargeted	Clinical	No

Lipid extraction

Extraction method	2-phase system	pH adjustment	None
2-phase system	Folch	Special conditions	Extraction done at lower temperatures
Were internal standards added prior extraction?	No		

Analytical platform

Ionizaton additives	Ammonium acetate	Number of separation dimensions	Two dimensions
Separation type 1	LC	Separation mode 1 (liquid)	RP
Separation window for lipid analyte selection (±) in minutes	2	Separation type 2	IMS
Separation mode 2 (generic)	Drift Tube (N2)	Detector	Mass spectrometer
MS type	QTOF	MS vendor	Agilent
Ion source	ESI	MS Level	MS^1, MS^2
Mass resolution for detected ion at MS^1	High resolution	Resolution at m/z 200 at MS^1	25000
Mass accuracy in ppm at MS^1	5	Recording mode of raw data at MS^1	Profile mode
Mass window for precursor ion isolation (in Da total isolation window)	0	Mass resolution for detected ion at MS^2	High resolution
Resolution at m/z 200 at MS^2	25000	Mass accuracy in ppm at MS^2	5
Recording mode of raw data at MS^2	Profile mode	Was/Were additional dimension/techniques used	Yes

Quality control

Blanks	Yes	Type of Blanks	Extraction blank, Solvent blank
Quality control	Yes	Type of QC sample	Commercial sample, Reference material

Method qualification and validation

Method validation	No

Reporting

Are reported raw data uploaded into repository?	Yes	Link to repository / ID to entry	https://doi.org/doi:10.25345/C5TT4G63P
Are metadata available?	Available on request	Summary data	Identification data
Raw data upload	Yes	Additional comments	All-ions fragmentation was performed after the IMS separation.

Sample Descriptions

Human Serum PFAS/Lipids / Human / Serum

Storage and collection conditions	Available	Temperature handling original sample	Unknown
Instant sample preparation	No	Storage temperature	-80 °C
Additives	None		

Lipid Class Descriptions

1) Cer[M-H]- / Lipid identification

Lipid class	Cer	MS Level for identification	MS^1, MS^2
Identification level	sn Position	MS^1 adduct	[M-H]-
Isotope correction at MS^1	No	MS^2 adduct	[M-H]-

Fragments for identification

Fragment name
FA1(+C2H3N)
FA1(+HN)
FA1(+C2H3NO)
LCB(-H6NO)
LCB(-CH3O)
LCB(-C2H8NO)

Isotope correction at MS^2	No	MS^1 verified by standard	No
MS^2 verified by standard	No	Background check at MS^1	No
Background check at MS^2	No	Did you presume assumptions for identification?	No
Limit of detection	No	RT verified by standard	No
Separation of isobaric/isomeric interferece confirmed	No	Model for separation prediction	No
Additional dimension/techniques	IMS	CCS verified by standard	No
How was/were the additional dimension(s) used?	For separation of isobaric/isomeric interference in MS1 and MS2 dimensions	Was a model used to predict lipid molecule separation?	No
Lipid Identification Software	Skyline	Nomenclature for intact lipid molecule	Yes
Nomenclature for fragment ions	Yes		

1) Cer[M-H]- / Lipid quantification

Quantitative	No	Normalization to reference	No
Batch correction	No		

2) Cer[M+HCOO]- / Lipid identification

Lipid class	Cer	MS Level for identification	MS^1, MS^2
Identification level	sn Position	MS^1 adduct	[M+HCOO]-
Isotope correction at MS^1	No	MS^2 adduct	[M-H]-

Fragments for identification

Fragment name
FA1(+C2H3N)
FA1(+HN)
FA1(+C2H3NO)
LCB(-H6NO)
LCB(-CH3O)
LCB(-C2H8NO)

Isotope correction at MS^2	No	MS^1 verified by standard	No
MS^2 verified by standard	No	Background check at MS^1	No

Background check at MS^2	No	Did you presume assumptions for identification?	No
Limit of detection	No	RT verified by standard	No
Separation of isobaric/isomeric interferece confirmed	No	Model for separation prediction	No
Additional dimension/techniques	IMS	CCS verified by standard	No
How was/were the additional dimension(s) used?	For separation of isobaric/isomeric interference in MS1 and MS2 dimensions	Was a model used to predict lipid molecule separation?	No
Lipid Identification Software	Skyline	Nomenclature for intact lipid molecule	Yes
Nomenclature for fragment ions	Yes		

2) Cer[M+HCOO]- / Lipid quantification

Quantitative	No	Normalization to reference	No
Batch correction	No		

3) Cer[M+CH3COO]- / Lipid identification

Lipid class	Cer	MS Level for identification	MS^1, MS^2
Identification level	sn Position	MS^1 adduct	[M+CH3COO]-
Isotope correction at MS^1	No	MS^2 adduct	[M-H]-

Fragments for identification

Fragment name
FA1(+C2H3N)
FA1(+HN)
FA1(+C2H3NO)
LCB(-H6NO)
LCB(-CH3O)
LCB(-C2H8NO)

Isotope correction at MS^2	No	MS^1 verified by standard	No
MS^2 verified by standard	No	Background check at MS^1	No
Background check at MS^2	No	Did you presume assumptions for identification?	No
Limit of detection	No	RT verified by standard	No
Separation of isobaric/isomeric interferece confirmed	No	Model for separation prediction	No
Additional dimension/techniques	IMS	CCS verified by standard	No
How was/were the additional dimension(s) used?	For separation of isobaric/isomeric interference in MS1 and MS2 dimensions	Was a model used to predict lipid molecule separation?	No
Lipid Identification Software	Skyline	Nomenclature for intact lipid molecule	Yes
Nomenclature for fragment ions	Yes		

3) Cer[M+CH3COO]- / Lipid quantification

Quantitative	No	Normalization to reference	No
Batch correction	No		

4) CL[M-H]- / Lipid identification

Lipid class	CL	MS Level for identification	MS^1
Identification level	Species level	MS^1 adduct	[M-H]-
Isotope correction at MS^1	No	MS^1 verified by standard	No
Background check at MS^1	No	Did you presume assumptions for identification?	No
Limit of detection	No	RT verified by standard	No
Separation of isobaric/isomeric interferece confirmed	No	Model for separation prediction	No
Additional dimension/techniques	IMS	CCS verified by standard	No
How was/were the additional dimension(s) used?	For separation of isobaric/isomeric interference in MS1 and MS2 dimensions	Was a model used to predict lipid molecule separation?	No
Lipid Identification Software	Skyline	Nomenclature for intact lipid molecule	Yes

4) CL[M-H]- / Lipid quantification

Quantitative	No	Normalization to reference	No
Batch correction	No		

5) CL[M-2H]2- / Lipid identification

Lipid class	CL	MS Level for identification	MS^1
Identification level	Species level	MS^1 adduct	[M-2H]2-
Isotope correction at MS^1	No	MS^1 verified by standard	No
Background check at MS^1	No	Did you presume assumptions for identification?	No
Limit of detection	No	RT verified by standard	No
Separation of isobaric/isomeric interferece confirmed	No	Model for separation prediction	No
Additional dimension/techniques	IMS	CCS verified by standard	No
How was/were the additional dimension(s) used?	For separation of isobaric/isomeric interference in MS1 and MS2 dimensions	Was a model used to predict lipid molecule separation?	No
Lipid Identification Software	Skyline	Nomenclature for intact lipid molecule	Yes

5) CL[M-2H]2- / Lipid quantification

Quantitative	No	Normalization to reference	No
Batch correction	No		

6) FA[M-H]- / Lipid identification

Lipid class	FA	MS Level for identification	MS^1
Identification level	Species level	MS^1 adduct	[M-H]-
Isotope correction at MS^1	No	MS^1 verified by standard	No
Background check at MS^1	No	Did you presume assumptions for identification?	No
Limit of detection	No	RT verified by standard	No
Separation of isobaric/isomeric interferece confirmed	No	Model for separation prediction	No
Additional dimension/techniques	IMS	CCS verified by standard	No
How was/were the additional dimension(s) used?	For separation of isobaric/isomeric interference in MS1 and MS2 dimensions	Was a model used to predict lipid molecule separation?	No
Lipid Identification Software	Skyline	Nomenclature for intact lipid molecule	Yes

6) FA[M-H]- / Lipid quantification

Quantitative	No	Normalization to reference	No
Batch correction	No		

7) FAHFA[M-H]- / Lipid identification

Lipid class	FAHFA	MS Level for identification	MS^1, MS^2
Identification level	sn Position	MS^1 adduct	[M-H]-
Isotope correction at MS^1	No	MS^2 adduct	[M-H]-

Fragments for identification

Fragment name
FA1(-H)

Isotope correction at MS^2	No	MS^1 verified by standard	No
MS^2 verified by standard	No	Background check at MS^1	No
Background check at MS^2	No	Did you presume assumptions for identification?	No
Limit of detection	No	RT verified by standard	No
Separation of isobaric/isomeric interferece confirmed	No	Model for separation prediction	No
Additional dimension/techniques	IMS	CCS verified by standard	No
How was/were the additional dimension(s) used?	For separation of isobaric/isomeric interference in MS1 and MS2 dimensions	Was a model used to predict lipid molecule separation?	No
Lipid Identification Software	Skyline	Nomenclature for intact lipid molecule	Yes
Nomenclature for fragment ions	Yes		

7) FAHFA[M-H]- / Lipid quantification

Quantitative	No	Normalization to reference	No
Batch correction	No		

8) GM3[M-H]- / Lipid identification

Lipid class	GM3	MS Level for identification	MS^1, MS^2
Identification level	sn Position	MS^1 adduct	[M-H]-
Isotope correction at MS^1	No	MS^2 adduct	[M-H]-

Fragments for identification

Fragment name
HG(NHex,290)
-HG(NHex,291)
-HG(NHex2,453)
-HG(NHex2,471)
-HG(NHex3,615)
-HG(NHex3,633)

Isotope correction at MS^2	No	MS^1 verified by standard	No
MS^2 verified by standard	No	Background check at MS^1	No
Background check at MS^2	No	Did you presume assumptions for identification?	No
Limit of detection	No	RT verified by standard	No
Separation of isobaric/isomeric interferece confirmed	No	Model for separation prediction	No
Additional dimension/techniques	IMS	CCS verified by standard	No
How was/were the additional dimension(s) used?	For separation of isobaric/isomeric interference in MS1 and MS2 dimensions	Was a model used to predict lipid molecule separation?	No
Lipid Identification Software	Skyline	Nomenclature for intact lipid molecule	Yes
Nomenclature for fragment ions	Yes		

8) GM3[M-H]- / Lipid quantification

Quantitative	No	Normalization to reference	No
Batch correction	No		

9) GD1[M-2H]2- / Lipid identification

Lipid class	GD1	MS Level for identification	MS^1
Identification level	Species level	MS^1 adduct	[M-2H]2-
Isotope correction at MS^1	No	MS^1 verified by standard	No
Background check at MS^1	No	Did you presume assumptions for identification?	No
Limit of detection	No	RT verified by standard	No
Separation of isobaric/isomeric interferece confirmed	No	Model for separation prediction	No
Additional dimension/techniques	IMS	CCS verified by standard	No
How was/were the additional dimension(s) used?	For separation of isobaric/isomeric interference in MS1 and MS2 dimensions	Was a model used to predict lipid molecule separation?	No
Lipid Identification Software	Skyline	Nomenclature for intact lipid molecule	Yes

9) GD1[M-2H]2- / Lipid quantification

Quantitative	No	Normalization to reference	No
Batch correction	No		

10) GM1[M-2H]2- / Lipid identification

Lipid class	GM1	MS Level for identification	MS^1
Identification level	Species level	MS^1 adduct	[M-2H]2-
Isotope correction at MS^1	No	MS^1 verified by standard	No
Background check at MS^1	No	Did you presume assumptions for identification?	No
Limit of detection	No	RT verified by standard	No
Separation of isobaric/isomeric interferece confirmed	No	Model for separation prediction	No
Additional dimension/techniques	IMS	CCS verified by standard	No
How was/were the additional dimension(s) used?	For separation of isobaric/isomeric interference in MS1 and MS2 dimensions	Was a model used to predict lipid molecule separation?	No
Lipid Identification Software	Skyline	Nomenclature for intact lipid molecule	Yes

10) GM1[M-2H]2- / Lipid quantification

Quantitative	No	Normalization to reference	No
Batch correction	No		

11) HexCer[M-H]- / Lipid identification

Lipid class	HexCer	MS Level for identification	MS^1, MS^2
Identification level	sn Position	MS^1 adduct	[M-H]-
Isotope correction at MS^1	No	MS^2 adduct	[M-H]-

Fragments for identification

Fragment name
FA1(+C2H3N)
FA1(+C2H3NO)
FA1(+HN)
LCB(-C2H8NO)
LCB(-CH3O)
LCB(-H6NO)

Isotope correction at MS^2	No	MS^1 verified by standard	No
MS^2 verified by standard	No	Background check at MS^1	No
Background check at MS^2	No	Did you presume assumptions for identification?	No
Limit of detection	No	RT verified by standard	No
Separation of isobaric/isomeric interferece confirmed	No	Model for separation prediction	No
Additional dimension/techniques	IMS	CCS verified by standard	No

How was/were the additional dimension(s) used?	For separation of isobaric/isomeric interference in MS1 and MS2 dimensions	Was a model used to predict lipid molecule separation?	No
Lipid Identification Software	Skyline	Nomenclature for intact lipid molecule	Yes
Nomenclature for fragment ions	Yes		

11) HexCer[M-H]- / Lipid quantification

Quantitative	No	Normalization to reference	No
Batch correction	No		

12) HexCer[M+HCOO]- / Lipid identification

Lipid class	HexCer	MS Level for identification	MS^1, MS^2
Identification level	sn Position	MS^1 adduct	[M+HCOO]-
Isotope correction at MS^1	No	MS^2 adduct	[M-H]-

Fragments for identification

Fragment name
FA1(+C2H3N)
FA1(+C2H3NO)
FA1(+HN)
LCB(-C2H8NO)
LCB(-CH3O)
LCB(-H6NO)
-HG(Hex,162)
-HG(Hex,180)

Isotope correction at MS^2	No	MS^1 verified by standard	No
MS^2 verified by standard	No	Background check at MS^1	No
Background check at MS^2	No	Did you presume assumptions for identification?	No
Limit of detection	No	RT verified by standard	No
Separation of isobaric/isomeric interferece confirmed	No	Model for separation prediction	No
Additional dimension/techniques	IMS	CCS verified by standard	No
How was/were the additional dimension(s) used?	For separation of isobaric/isomeric interference in MS1 and MS2 dimensions	Was a model used to predict lipid molecule separation?	No
Lipid Identification Software	Skyline	Nomenclature for intact lipid molecule	Yes
Nomenclature for fragment ions	Yes		

12) HexCer[M+HCOO]- / Lipid quantification

Quantitative	No	Normalization to reference	No
Batch correction	No		

13) HexCer[M+CH3COO]- / Lipid identification

Lipid class	HexCer	MS Level for identification	MS^1, MS^2
Identification level	sn Position	MS^1 adduct	[M+CH3COO]-
Isotope correction at MS^1	No	MS^2 adduct	[M-H]-

Fragments for identification

Fragment name
FA1(+C2H3N)
FA1(+C2H3NO)
FA1(+HN)
LCB(-C2H8NO)
LCB(-CH3O)
LCB(-H6NO)
-HG(Hex,162)
-HG(Hex,180)

Isotope correction at MS^2	No	MS^1 verified by standard	No
MS^2 verified by standard	No	Background check at MS^1	No
Background check at MS^2	No	Did you presume assumptions for identification?	No
Limit of detection	No	RT verified by standard	No
Separation of isobaric/isomeric interferece confirmed	No	Model for separation prediction	No
Additional dimension/techniques	IMS	CCS verified by standard	No
How was/were the additional dimension(s) used?	For separation of isobaric/isomeric interference in MS1 and MS2 dimensions	Was a model used to predict lipid molecule separation?	No
Lipid Identification Software	Skyline	Nomenclature for intact lipid molecule	Yes
Nomenclature for fragment ions	Yes		

13) HexCer[M+CH3COO]- / Lipid quantification

Quantitative	No	Normalization to reference	No
Batch correction	No		

14) LPC[M+CH3COO]- / Lipid identification

Lipid class	LPC	MS Level for identification	MS^1, MS^2
Identification level	sn Position	MS^1 adduct	[M+CH3COO]-
Isotope correction at MS^1	No	MS^2 adduct	[M-H]-

Fragments for identification

Fragment name
HG(PC,224)
FA1(+O)
-(CH3+CH3COO)

Isotope correction at MS^2	No	MS^1 verified by standard	No
MS^2 verified by standard	No	Background check at MS^1	No
Background check at MS^2	No	Did you presume assumptions for identification?	Yes

Which assumptions were presumed?	Elution order is LPC(0:0/x:x) followed by LPC(x:x/0:0)	Limit of detection	No
RT verified by standard	No	Separation of isobaric/isomeric interferece confirmed	No
Model for separation prediction	No	Additional dimension/techniques	IMS
CCS verified by standard	No	How was/were the additional dimension(s) used?	For separation of isobaric/isomeric interference in MS1 and MS2 dimensions
Was a model used to predict lipid molecule separation?	No	Lipid Identification Software	Skyline
Nomenclature for intact lipid molecule	Yes	Nomenclature for fragment ions	Yes

14) LPC[M+CH3COO]- / Lipid quantification

Quantitative	No	Normalization to reference	No
Batch correction	No		

15) LPE[M-H]- / Lipid identification

Lipid class	LPE	MS Level for identification	MS^1, MS^2
Identification level	sn Position	MS^1 adduct	[M-H]-
Isotope correction at MS^1	No	MS^2 adduct	[M-H]-

Fragments for identification

Fragment name
-FA1(-H)
-FA1(-H)-(H2O)
FA1(+O)
GP(153)

Isotope correction at MS^2	No	MS^1 verified by standard	No
MS^2 verified by standard	No	Background check at MS^1	No
Background check at MS^2	No	Did you presume assumptions for identification?	Yes
Which assumptions were presumed?	Elution order is LPE(0:0/x:x) followed by LPE(x:x/0:0)	Limit of detection	No
RT verified by standard	No	Separation of isobaric/isomeric interferece confirmed	No
Model for separation prediction	No	Additional dimension/techniques	IMS
CCS verified by standard	No	How was/were the additional dimension(s) used?	For separation of isobaric/isomeric interference in MS1 and MS2 dimensions
Was a model used to predict lipid molecule separation?	No	Lipid Identification Software	Skyline
Nomenclature for intact lipid molecule	Yes	Nomenclature for fragment ions	Yes

15) LPE[M-H]- / Lipid quantification

Quantitative	No	Normalization to reference	No
Batch correction	No		

16) LPE O[M-H]- / Lipid identification

Lipid class	LPE O	MS Level for identification	MS^1, MS^2
Identification level	sn Position	MS^1 adduct	[M-H]-
Isotope correction at MS^1	No	MS^2 adduct	[M-H]-

Fragments for identification

Fragment name
-FA1(-H)
GP(153)

Isotope correction at MS^2	No	MS^1 verified by standard	No
MS^2 verified by standard	No	Background check at MS^1	No
Background check at MS^2	No	Did you presume assumptions for identification?	No
Limit of detection	No	RT verified by standard	No
Separation of isobaric/isomeric interferece confirmed	No	Model for separation prediction	No
Additional dimension/techniques	IMS	CCS verified by standard	No
How was/were the additional dimension(s) used?	For separation of isobaric/isomeric interference in MS1 and MS2 dimensions	Was a model used to predict lipid molecule separation?	No
Lipid Identification Software	Skyline	Nomenclature for intact lipid molecule	Yes
Nomenclature for fragment ions	Yes		

16) LPE O[M-H]- / Lipid quantification

Quantitative	No	Normalization to reference	No
Batch correction	No		

17) LPG[M-H]- / Lipid identification

Lipid class	LPG	MS Level for identification	MS^1, MS^2
Identification level	sn Position	MS^1 adduct	[M-H]-
Isotope correction at MS^1	No	MS^2 adduct	[M-H]-

Fragments for identification

Fragment name
-FA1(-H)
-FA1(-H)-(H2O)
FA1(+O)
GP(153)
P(79)

Isotope correction at MS^2	No	MS^1 verified by standard	No
MS^2 verified by standard	No	Background check at MS^1	No
Background check at MS^2	No	Did you presume assumptions for identification?	Yes

Which assumptions were presumed?	Elution order is LPG(0:0/x:x) followed by LPG(x:x/0:0)	Limit of detection	No
RT verified by standard	No	Separation of isobaric/isomeric interferece confirmed	No
Model for separation prediction	No	Additional dimension/techniques	IMS
CCS verified by standard	No	How was/were the additional dimension(s) used?	For separation of isobaric/isomeric interference in MS1 and MS2 dimensions
Was a model used to predict lipid molecule separation?	No	Lipid Identification Software	Skyline
Nomenclature for intact lipid molecule	Yes	Nomenclature for fragment ions	Yes

17) LPG[M-H]- / Lipid quantification

Quantitative	No	Normalization to reference	No
Batch correction	No		

18) LPI[M-H]- / Lipid identification

Lipid class	LPI	MS Level for identification	MS^1, MS^2
Identification level	sn Position	MS^1 adduct	[M-H]-
Isotope correction at MS^1	No	MS^2 adduct	[M-H]-

Fragments for identification

Fragment name
-(C6H12O6, 180)
-FA1(+HO)
FA1(+O)
GP(153)
HG(PI,241)
P(79)

Isotope correction at MS^2	No	MS^1 verified by standard	No
MS^2 verified by standard	No	Background check at MS^1	No
Background check at MS^2	No	Did you presume assumptions for identification?	Yes
Which assumptions were presumed?	Elution order is LPI(0:0/x:x) followed by LPI(x:x/0:0)	Limit of detection	No
RT verified by standard	No	Separation of isobaric/isomeric interferece confirmed	No
Model for separation prediction	No	Additional dimension/techniques	IMS
CCS verified by standard	No	How was/were the additional dimension(s) used?	For separation of isobaric/isomeric interference in MS1 and MS2 dimensions
Was a model used to predict lipid molecule separation?	No	Lipid Identification Software	Skyline
Nomenclature for intact lipid molecule	Yes	Nomenclature for fragment ions	Yes

18) LPI[M-H]- / Lipid quantification

Quantitative	No	Normalization to reference	No
Batch correction	No		

19) PA[M-H]- / Lipid identification

Lipid class	PA	MS Level for identification	MS^1, MS^2
Identification level	Molecular species level	MS^1 adduct	[M-H]-
Isotope correction at MS^1	No	MS^2 adduct	[M-H]-

Fragments for identification

Fragment name
FA1(+O)
FA2(+O)
-FA1(+HO)
-FA2(+HO)
-FA1(-H)
-FA2(-H)
GP(153)

Isotope correction at MS^2	No	MS^1 verified by standard	No
MS^2 verified by standard	No	Background check at MS^1	No
Background check at MS^2	No	Did you presume assumptions for identification?	No
Limit of detection	No	RT verified by standard	No
Separation of isobaric/isomeric interference confirmed	No	Model for separation prediction	No
Additional dimension/techniques	IMS	CCS verified by standard	No
How was/were the additional dimension(s) used?	For separation of isobaric/isomeric interference in MS1 and MS2 dimensions	Was a model used to predict lipid molecule separation?	No
Lipid Identification Software	Skyline	Nomenclature for intact lipid molecule	Yes
Nomenclature for fragment ions	Yes		

19) PA[M-H]- / Lipid quantification

Quantitative	No	Normalization to reference	No
Batch correction	No		

20) PC[M+CH3COO]- / Lipid identification

Lipid class	PC	MS Level for identification	MS^1, MS^2
Identification level	Molecular species level	MS^1 adduct	[M+CH3COO]-
Isotope correction at MS^1	No	MS^2 adduct	[M-H]-

Fragments for identification

Fragment name
-(CH3+CH3COO)
-FA1(+HO)-(CH3+CH3COO)
-FA2(+HO)-(CH3+CH3COO)
-FA1(-H)-(CH3+CH3COO)

Fragment name
-FA2(-H)-(CH3+CH3COO)
FA1(+O)
FA2(+O)
HG(PC,224)

Isotope correction at MS^2	No	MS^1 verified by standard	No
MS^2 verified by standard	No	Background check at MS^1	No
Background check at MS^2	No	Did you presume assumptions for identification?	No
Limit of detection	No	RT verified by standard	No
Separation of isobaric/isomeric interferece confirmed	No	Model for separation prediction	No
Additional dimension/techniques	IMS	CCS verified by standard	No
How was/were the additional dimension(s) used?	For separation of isobaric/isomeric interference in MS1 and MS2 dimensions	Was a model used to predict lipid molecule separation?	No
Lipid Identification Software	Skyline	Nomenclature for intact lipid molecule	Yes
Nomenclature for fragment ions	Yes		

20) PC[M+CH3COO]- / Lipid quantification

Quantitative	No	Normalization to reference	No
Batch correction	No		

21) PC O[M+CH3COO]- / Lipid identification

Lipid class	PC O	MS Level for identification	MS^1, MS^2
Identification level	sn Position	MS^1 adduct	[M+CH3COO]-
Isotope correction at MS^1	No	MS^2 adduct	[M-H]-

Fragments for identification

Fragment name
-(CH3+CH3COO)
-FA2(+HO)
-FA2(-H)
FA2 -(CO)
FA2(+O)
FA1
HG(PC)-(CH3+CH3COO)

Isotope correction at MS^2	No	MS^1 verified by standard	No
MS^2 verified by standard	No	Background check at MS^1	No
Background check at MS^2	No	Did you presume assumptions for identification?	No
Limit of detection	No	RT verified by standard	No
Separation of isobaric/isomeric interferece confirmed	No	Model for separation prediction	No
Additional dimension/techniques	IMS	CCS verified by standard	No
How was/were the additional dimension(s) used?	For separation of isobaric/isomeric interference in MS1 and MS2 dimensions	Was a model used to predict lipid molecule separation?	No
Lipid Identification Software	Skyline	Nomenclature for intact lipid molecule	Yes

Nomenclature for fragment ions	Yes		

21) PC O[M+CH3COO]- / Lipid quantification

Quantitative	No	Normalization to reference	No
Batch correction	No		

22) PC P[M+CH3COO]- / Lipid identification

Lipid class	PC P	MS Level for identification	MS^1, MS^2
Identification level	sn Position	MS^1 adduct	[M+CH3COO]-
Isotope correction at MS^1	No	MS^2 adduct	[M-H]-

Fragments for identification

Fragment name
-(CH3+CH3COO)
-FA2(+HO)
-FA2(-H)
FA2 -(CO)
FA2(+O)
FA1
HG(PC)-(CH3+CH3COO)

Isotope correction at MS^2	No	MS^1 verified by standard	No
MS^2 verified by standard	No	Background check at MS^1	No
Background check at MS^2	No	Did you presume assumptions for identification?	No
Limit of detection	No	RT verified by standard	No
Separation of isobaric/isomeric interferece confirmed	No	Model for separation prediction	No
Additional dimension/techniques	IMS	CCS verified by standard	No
How was/were the additional dimension(s) used?	For separation of isobaric/isomeric interference in MS1 and MS2 dimensions	Was a model used to predict lipid molecule separation?	No
Lipid Identification Software	Skyline	Nomenclature for intact lipid molecule	Yes
Nomenclature for fragment ions	Yes		

22) PC P[M+CH3COO]- / Lipid quantification

Quantitative	No	Normalization to reference	No
Batch correction	No		

23) PE[M-H]- / Lipid identification

Lipid class	PE	MS Level for identification	MS^1, MS^2
Identification level	Molecular species level	MS^1 adduct	[M-H]-

Isotope correction at MS^1	No	MS^2 adduct	[M-H]-

Fragments for identification

Fragment name
HG(PE,196)
GP(153)
FA1(+O)
FA2(+O)
-FA1(+HO)
-FA2(+HO)
-FA1(-H)
-FA2(-H)

Isotope correction at MS^2	No	MS^1 verified by standard	No
MS^2 verified by standard	No	Background check at MS^1	No
Background check at MS^2	No	Did you presume assumptions for identification?	No
Limit of detection	No	RT verified by standard	No
Separation of isobaric/isomeric interferece confirmed	No	Model for separation prediction	No
Additional dimension/techniques	IMS	CCS verified by standard	No
How was/were the additional dimension(s) used?	For separation of isobaric/isomeric interference in MS1 and MS2 dimensions	Was a model used to predict lipid molecule separation?	No
Lipid Identification Software	Skyline	Nomenclature for intact lipid molecule	Yes
Nomenclature for fragment ions	Yes		

23) PE[M-H]- / Lipid quantification

Quantitative	No	Normalization to reference	No
Batch correction	No		

24) PE O[M-H]- / Lipid identification

Lipid class	PE O	MS Level for identification	MS^1, MS^2
Identification level	sn Position	MS^1 adduct	[M-H]-
Isotope correction at MS^1	No	MS^2 adduct	[M-H]-

Fragments for identification

Fragment name
FA2(+O)
-FA2(+HO)
-FA2(-H)
FA2 -(CO)
GP(135)
GP(153)

Isotope correction at MS^2	No	MS^1 verified by standard	No
MS^2 verified by standard	No	Background check at MS^1	No
Background check at MS^2	No	Did you presume assumptions for identification?	No
Limit of detection	No	RT verified by standard	No

Separation of isobaric/isomeric interferece confirmed	No	Model for separation prediction	No
Additional dimension/techniques	IMS	CCS verified by standard	No
How was/were the additional dimension(s) used?	For separation of isobaric/isomeric interference in MS1 and MS2 dimensions	Was a model used to predict lipid molecule separation?	No
Lipid Identification Software	Skyline	Nomenclature for intact lipid molecule	Yes
Nomenclature for fragment ions	Yes		

24) PE O[M-H]- / Lipid quantification

Quantitative	No	Normalization to reference	No
Batch correction	No		

25) PE P[M-H]- / Lipid identification

Lipid class	PE P	MS Level for identification	MS^1, MS^2
Identification level	sn Position	MS^1 adduct	[M-H]-
Isotope correction at MS^1	No	MS^2 adduct	[M-H]-

Fragments for identification

Fragment name
FA2(+O)
-FA2(+HO)
-FA2(-H)
FA2 -(CO)
FA1
HG(PE,196)

Isotope correction at MS^2	No	MS^1 verified by standard	No
MS^2 verified by standard	No	Background check at MS^1	No
Background check at MS^2	No	Did you presume assumptions for identification?	No
Limit of detection	No	RT verified by standard	No
Separation of isobaric/isomeric interferece confirmed	No	Model for separation prediction	No
Additional dimension/techniques	IMS	CCS verified by standard	No
How was/were the additional dimension(s) used?	For separation of isobaric/isomeric interference in MS1 and MS2 dimensions	Was a model used to predict lipid molecule separation?	No
Lipid Identification Software	Skyline	Nomenclature for intact lipid molecule	Yes
Nomenclature for fragment ions	Yes		

25) PE P[M-H]- / Lipid quantification

Quantitative	No	Normalization to reference	No
Batch correction	No		

26) PG[M-H]- / Lipid identification

Lipid class	PG	MS Level for identification	MS^1, MS^2
Identification level	Molecular species level	MS^1 adduct	[M-H]-
Isotope correction at MS^1	No	MS^2 adduct	[M-H]-

Fragments for identification

Fragment name
-FA1(+HO)
-FA1(-H)
FA1(+O)
-FA2(+HO)
-FA2(-H)
FA2(+O)
GP(153)
HG(PG,171)
HG(PG,227)

Isotope correction at MS^2	No	MS^1 verified by standard	No
MS^2 verified by standard	No	Background check at MS^1	No
Background check at MS^2	No	Did you presume assumptions for identification?	No
Limit of detection	No	RT verified by standard	No
Separation of isobaric/isomeric interferece confirmed	No	Model for separation prediction	No
Additional dimension/techniques	IMS	CCS verified by standard	No
How was/were the additional dimension(s) used?	For separation of isobaric/isomeric interference in MS1 and MS2 dimensions	Was a model used to predict lipid molecule separation?	No
Lipid Identification Software	Skyline	Nomenclature for intact lipid molecule	Yes
Nomenclature for fragment ions	Yes		

26) PG[M-H]- / Lipid quantification

Quantitative	No	Normalization to reference	No
Batch correction	No		

27) PI[M-H]- / Lipid identification

Lipid class	PI	MS Level for identification	MS^1, MS^2
Identification level	Molecular species level	MS^1 adduct	[M-H]-
Isotope correction at MS^1	No	MS^2 adduct	[M-H]-

Fragments for identification

Fragment name
-FA1(-H)
FA1(+O)
-FA1(+HO)
-FA2(-H)
FA2(+O)
-FA2(+HO)

Fragment name
GP(153)
HG(PI,241)

Isotope correction at MS^2	No	MS^1 verified by standard	No
MS^2 verified by standard	No	Background check at MS^1	No
Background check at MS^2	No	Did you presume assumptions for identification?	No
Limit of detection	No	RT verified by standard	No
Separation of isobaric/isomeric interferece confirmed	No	Model for separation prediction	No
Additional dimension/techniques	IMS	CCS verified by standard	No
How was/were the additional dimension(s) used?	For separation of isobaric/isomeric interference in MS1 and MS2 dimensions	Was a model used to predict lipid molecule separation?	No
Lipid Identification Software	Skyline	Nomenclature for intact lipid molecule	Yes
Nomenclature for fragment ions	Yes		

27) PI[M-H]- / Lipid quantification

Quantitative	No	Normalization to reference	No
Batch correction	No		

28) PS[M-H]- / Lipid identification

Lipid class	PS	MS Level for identification	MS^1, MS^2
Identification level	Molecular species level	MS^1 adduct	[M-H]-
Isotope correction at MS^1	No	MS^2 adduct	[M-H]-

Fragments for identification

Fragment name
-(C3H5NO2,87)
-FA1(+HO)-(C3H5NO2)
-FA1(-H)-(C3H5NO2)
FA1(+O)
-FA2(+HO)-(C3H5NO2)
-FA2(-H)-(C3H5NO2)
FA2(+O)
GP(153)

Isotope correction at MS^2	No	MS^1 verified by standard	No
MS^2 verified by standard	No	Background check at MS^1	No
Background check at MS^2	No	Did you presume assumptions for identification?	No
Limit of detection	No	RT verified by standard	No
Separation of isobaric/isomeric interferece confirmed	No	Model for separation prediction	No
Additional dimension/techniques	IMS	CCS verified by standard	No
How was/were the additional dimension(s) used?	For separation of isobaric/isomeric interference in MS1 and MS2 dimensions	Was a model used to predict lipid molecule separation?	No
Lipid Identification Software	Skyline	Nomenclature for intact lipid molecule	Yes
Nomenclature for fragment ions	Yes		

28) PS[M-H]- / Lipid quantification

Quantitative	No	Normalization to reference	No
Batch correction	No		

29) SM[M+HCOO]- / Lipid identification

Lipid class	SM	MS Level for identification	MS^1, MS^2
Identification level	sn Position	MS^1 adduct	[M+HCOO]-
Isotope correction at MS^1	No	MS^2 adduct	[M-H]-
Fragments for identification			

Fragment name
FA1(+O)
HG(PC,168)

Isotope correction at MS^2	No	MS^1 verified by standard	No
MS^2 verified by standard	No	Background check at MS^1	No
Background check at MS^2	No	Did you presume assumptions for identification?	No
Limit of detection	No	RT verified by standard	No
Separation of isobaric/isomeric interferece confirmed	No	Model for separation prediction	No
Additional dimension/techniques	IMS	CCS verified by standard	No
How was/were the additional dimension(s) used?	For separation of isobaric/isomeric interference in MS1 and MS2 dimensions	Was a model used to predict lipid molecule separation?	No
Lipid Identification Software	Skyline	Nomenclature for intact lipid molecule	Yes
Nomenclature for fragment ions	Yes		

29) SM[M+HCOO]- / Lipid quantification

Quantitative	No	Normalization to reference	No
Batch correction	No		

30) SM[M+CH3COO]- / Lipid identification

Lipid class	SM	MS Level for identification	MS^1, MS^2
Identification level	sn Position	MS^1 adduct	[M+CH3COO]-
Isotope correction at MS^1	No	MS^2 adduct	[M-H]-
Fragments for identification			

Fragment name
FA1(+O)
HG(PC,168)

Isotope correction at MS^2	No	MS^1 verified by standard	No
MS^2 verified by standard	No	Background check at MS^1	No
Background check at MS^2	No	Did you presume assumptions for identification?	No
Limit of detection	No	RT verified by standard	No
Separation of isobaric/isomeric interferece confirmed	No	Model for separation prediction	No
Additional dimension/techniques	IMS	CCS verified by standard	No
How was/were the additional dimension(s) used?	For separation of isobaric/isomeric interference in MS1 and MS2 dimensions	Was a model used to predict lipid molecule separation?	No
Lipid Identification Software	Skyline	Nomenclature for intact lipid molecule	Yes
Nomenclature for fragment ions	Yes		

30) SM[M+CH3COO]- / Lipid quantification

Quantitative	No	Normalization to reference	No
Batch correction	No		

31) CAR[M+H]+ / Lipid identification

Lipid class	CAR	MS Level for identification	MS^1, MS^2
Identification level	Molecular species level	MS^1 adduct	[M+H]+
Isotope correction at MS^1	No	MS^2 adduct	[M+H]+

Fragments for identification

Fragment name
M-FA-TMA

Isotope correction at MS^2	No	MS^1 verified by standard	No
MS^2 verified by standard	No	Background check at MS^1	No
Background check at MS^2	No	Did you presume assumptions for identification?	No
Limit of detection	No	RT verified by standard	No
Separation of isobaric/isomeric interferece confirmed	No	Model for separation prediction	No
Additional dimension/techniques	IMS	CCS verified by standard	No
How was/were the additional dimension(s) used?	For separation of isobaric/isomeric interference in MS1 and MS2 dimensions	Was a model used to predict lipid molecule separation?	No
Lipid Identification Software	Skyline	Nomenclature for intact lipid molecule	Yes
Nomenclature for fragment ions	Yes		

31) CAR[M+H]+ / Lipid quantification

Quantitative	No	Normalization to reference	No
Batch correction	No		

32) NAE[M+H]+ / Lipid identification

Lipid class	NAE	MS Level for identification	MS^1
Identification level	Species level	MS^1 adduct	[M+H]+
Isotope correction at MS^1	No	MS^1 verified by standard	No
Background check at MS^1	No	Did you presume assumptions for identification?	No
Limit of detection	No	RT verified by standard	No
Separation of isobaric/isomeric interferece confirmed	No	Model for separation prediction	No
Additional dimension/techniques	IMS	CCS verified by standard	No
How was/were the additional dimension(s) used?	For separation of isobaric/isomeric interference in MS1 and MS2 dimensions	Was a model used to predict lipid molecule separation?	No
Lipid Identification Software	Skyline	Nomenclature for intact lipid molecule	No

32) NAE[M+H]+ / Lipid quantification

Quantitative	No	Normalization to reference	No
Batch correction	No		

33) CE[M+NH4]+ / Lipid identification

Lipid class	CE	MS Level for identification	MS^1, MS^2
Identification level	Species level	MS^1 adduct	[M+NH4]+
Isotope correction at MS^1	No	MS^2 adduct	[M+H]+

Fragments for identification

Fragment name
-FA1(+HO)-Cholesterol(35)

Isotope correction at MS^2	No	MS^1 verified by standard	No
MS^2 verified by standard	No	Background check at MS^1	No
Background check at MS^2	No	Did you presume assumptions for identification?	No
Limit of detection	No	RT verified by standard	No
Separation of isobaric/isomeric interferece confirmed	No	Model for separation prediction	No
Additional dimension/techniques	IMS	CCS verified by standard	No
How was/were the additional dimension(s) used?	For separation of isobaric/isomeric interference in MS1 and MS2 dimensions	Was a model used to predict lipid molecule separation?	No
Lipid Identification Software	Skyline	Nomenclature for intact lipid molecule	Yes
Nomenclature for fragment ions	Yes		

33) CE[M+NH4]+ / Lipid quantification

Quantitative	No	Normalization to reference	No
Batch correction	No		

34) Cer[M+H]+ / Lipid identification

Lipid class	Cer	MS Level for identification	MS^1, MS^2
Identification level	sn Position	MS^1 adduct	[M+H]+
Isotope correction at MS^1	No	MS^2 adduct	[M+H]+

Fragments for identification

Fragment name
LCB(-HO)
LCB(-H3O2)
LCB(-CH3O2)

Isotope correction at MS^2	No	MS^1 verified by standard	No
MS^2 verified by standard	No	Background check at MS^1	No
Background check at MS^2	No	Did you presume assumptions for identification?	No
Limit of detection	No	RT verified by standard	No
Separation of isobaric/isomeric interference confirmed	No	Model for separation prediction	No
Additional dimension/techniques	IMS	CCS verified by standard	No
How was/were the additional dimension(s) used?	For separation of isobaric/isomeric interference in MS1 and MS2 dimensions	Was a model used to predict lipid molecule separation?	No
Lipid Identification Software	Skyline	Nomenclature for intact lipid molecule	Yes
Nomenclature for fragment ions	Yes		

34) Cer[M+H]+ / Lipid quantification

Quantitative	No	Normalization to reference	No
Batch correction	No		

35) DG[M+NH4]+ / Lipid identification

Lipid class	DG	MS Level for identification	MS^1, MS^2
Identification level	Molecular species level	MS^1 adduct	[M+NH4]+
Isotope correction at MS^1	No	MS^2 adduct	[M+H]+

Fragments for identification

Fragment name
-FA1(-H)-(H2O+NH3)
-FA2(-H)-(H2O+NH3)

Isotope correction at MS^2	No	MS^1 verified by standard	No
MS^2 verified by standard	No	Background check at MS^1	No
Background check at MS^2	No	Did you presume assumptions for identification?	No
Limit of detection	No	RT verified by standard	No
Separation of isobaric/isomeric interference confirmed	No	Model for separation prediction	No

Additional dimension/techniques	IMS	CCS verified by standard	No
How was/were the additional dimension(s) used?	For separation of isobaric/isomeric interference in MS1 and MS2 dimensions	Was a model used to predict lipid molecule separation?	No
Lipid Identification Software	Skyline	Nomenclature for intact lipid molecule	Yes
Nomenclature for fragment ions	Yes		

35) DG[M+NH4]+ / Lipid quantification

Quantitative	No	Normalization to reference	No
Batch correction	No		

36) LPC[M+H]+ / Lipid identification

Lipid class	LPC	MS Level for identification	MS^1, MS^2
Identification level	sn Position	MS^1 adduct	[M+H]+
Isotope correction at MS^1	No	MS^2 adduct	[M+H]+

Fragments for identification

Fragment name
(C5H13NO,104)

Isotope correction at MS^2	No	MS^1 verified by standard	No
MS^2 verified by standard	No	Background check at MS^1	No
Background check at MS^2	No	Did you presume assumptions for identification?	Yes
Which assumptions were presumed?	Elution order is LPC(0:0/x:x) followed by LPC(x:x/0:0)	Limit of detection	No
RT verified by standard	No	Separation of isobaric/isomeric interferece confirmed	No
Model for separation prediction	No	Additional dimension/techniques	IMS
CCS verified by standard	No	How was/were the additional dimension(s) used?	For separation of isobaric/isomeric interference in MS1 and MS2 dimensions
Was a model used to predict lipid molecule separation?	No	Lipid Identification Software	Skyline
Nomenclature for intact lipid molecule	Yes	Nomenclature for fragment ions	Yes

36) LPC[M+H]+ / Lipid quantification

Quantitative	No	Normalization to reference	No
Batch correction	No		

37) LPC[M+Na]+ / Lipid identification

Lipid class	LPC	MS Level for identification	MS^1, MS^2
Identification level	sn Position	MS^1 adduct	[M+Na]+

Isotope correction at MS^1	No	MS^2 adduct	[M+H]+

Fragments for identification

Fragment name
-HG(PC,183)
M+Na-TMA

Isotope correction at MS^2	No	MS^1 verified by standard	No
MS^2 verified by standard	No	Background check at MS^1	No
Background check at MS^2	No	Did you presume assumptions for identification?	Yes
Which assumptions were presumed?	Elution order is LPC(0:0/x:x) followed by LPC(x:x/0:0)	Limit of detection	No
RT verified by standard	No	Separation of isobaric/isomeric interferece confirmed	No
Model for separation prediction	No	Additional dimension/techniques	IMS
CCS verified by standard	No	How was/were the additional dimension(s) used?	For separation of isobaric/isomeric interference in MS1 and MS2 dimensions
Was a model used to predict lipid molecule separation?	No	Lipid Identification Software	Skyline
Nomenclature for intact lipid molecule	Yes	Nomenclature for fragment ions	Yes

37) LPC[M+Na]+ / Lipid quantification

Quantitative	No	Normalization to reference	No
Batch correction	No		

38) LPE[M+H]+ / Lipid identification

Lipid class	LPE	MS Level for identification	MS^1, MS^2
Identification level	sn Position	MS^1 adduct	[M+H]+
Isotope correction at MS^1	No	MS^2 adduct	[M+H]+

Fragments for identification

Fragment name
-HG(PE,141)

Isotope correction at MS^2	No	MS^1 verified by standard	No
MS^2 verified by standard	No	Background check at MS^1	No
Background check at MS^2	No	Did you presume assumptions for identification?	Yes
Which assumptions were presumed?	Elution order is LPE(0:0/x:x) followed by LPE(x:x/0:0)	Limit of detection	No
RT verified by standard	No	Separation of isobaric/isomeric interferece confirmed	No
Model for separation prediction	No	Additional dimension/techniques	IMS
CCS verified by standard	No	How was/were the additional dimension(s) used?	For separation of isobaric/isomeric interference in MS1 and MS2 dimensions
Was a model used to predict lipid molecule separation?	No	Lipid Identification Software	Skyline
Nomenclature for intact lipid molecule	Yes	Nomenclature for fragment ions	Yes

38) LPE[M+H]+ / Lipid quantification

Quantitative	No	Normalization to reference	No
Batch correction	No		

39) LPE[M+Na]+ / Lipid identification

Lipid class	LPE	MS Level for identification	MS^1, MS^2
Identification level	sn Position	MS^1 adduct	[M+Na]+
Isotope correction at MS^1	No	MS^2 adduct	[M+H]+

Fragments for identification

Fragment name
-HG(PE,141)
M+Na-az

Isotope correction at MS^2	No	MS^1 verified by standard	No
MS^2 verified by standard	No	Background check at MS^1	No
Background check at MS^2	No	Did you presume assumptions for identification?	Yes
Which assumptions were presumed?	Elution order is LPE(0:0/x:x) followed by LPE(x:x/0:0)	Limit of detection	No
RT verified by standard	No	Separation of isobaric/isomeric interferece confirmed	No
Model for separation prediction	No	Additional dimension/techniques	IMS
CCS verified by standard	No	How was/were the additional dimension(s) used?	For separation of isobaric/isomeric interference in MS1 and MS2 dimensions
Was a model used to predict lipid molecule separation?	No	Lipid Identification Software	Skyline
Nomenclature for intact lipid molecule	Yes	Nomenclature for fragment ions	Yes

39) LPE[M+Na]+ / Lipid quantification

Quantitative	No	Normalization to reference	No
Batch correction	No		

40) PC[M+H]+ / Lipid identification

Lipid class	PC	MS Level for identification	MS^1, MS^2
Identification level	Molecular species level	MS^1 adduct	[M+H]+
Isotope correction at MS^1	No	MS^2 adduct	[M+H]+

Fragments for identification

Fragment name
-FA1(+H)
-FA2(+H)

Isotope correction at MS^2	No	MS^1 verified by standard	No
MS^2 verified by standard	No	Background check at MS^1	No
Background check at MS^2	No	Did you presume assumptions for identification?	No
Limit of detection	No	RT verified by standard	No
Separation of isobaric/isomeric interferece confirmed	No	Model for separation prediction	No
Additional dimension/techniques	IMS	CCS verified by standard	No
How was/were the additional dimension(s) used?	For separation of isobaric/isomeric interference in MS1 and MS2 dimensions	Was a model used to predict lipid molecule separation?	No
Lipid Identification Software	Skyline	Nomenclature for intact lipid molecule	Yes
Nomenclature for fragment ions	Yes		

40) PC[M+H]+ / Lipid quantification

Quantitative	No	Normalization to reference	No
Batch correction	No		

41) PC[M+Na]+ / Lipid identification

Lipid class	PC	MS Level for identification	MS^1, MS^2
Identification level	Molecular species level	MS^1 adduct	[M+Na]+
Isotope correction at MS^1	No	MS^2 adduct	[M+H]+

Fragments for identification

Fragment name
M+Na-FA1
M+Na-FA2
M+Na-HG
M+Na-TMA
M+Na-TMA-FA1
M+Na-TMA-FA2
-FA1(+H)
-FA2(+H)
-HG(PC,183)
-TMA-FA1
-TMA-FA2

Isotope correction at MS^2	No	MS^1 verified by standard	No
MS^2 verified by standard	No	Background check at MS^1	No
Background check at MS^2	No	Did you presume assumptions for identification?	No
Limit of detection	No	RT verified by standard	No
Separation of isobaric/isomeric interferece confirmed	No	Model for separation prediction	No
Additional dimension/techniques	IMS	CCS verified by standard	No
How was/were the additional dimension(s) used?	For separation of isobaric/isomeric interference in MS1 and MS2 dimensions	Was a model used to predict lipid molecule separation?	No
Lipid Identification Software	Skyline	Nomenclature for intact lipid molecule	Yes
Nomenclature for fragment ions	Yes		

41) PC[M+Na]+ / Lipid quantification

Quantitative	No	Normalization to reference	No
Batch correction	No		

42) PC O[M+H]+ / Lipid identification

Lipid class	PC O	MS Level for identification	MS^1, MS^2
Identification level	sn Position	MS^1 adduct	[M+H]+
Isotope correction at MS^1	No	MS^2 adduct	[M+H]+

Fragments for identification

Fragment name
-FA1(+H)
-FA2(+H)

Isotope correction at MS^2	No	MS^1 verified by standard	No
MS^2 verified by standard	No	Background check at MS^1	No
Background check at MS^2	No	Did you presume assumptions for identification?	No
Limit of detection	No	RT verified by standard	No
Separation of isobaric/isomeric interferece confirmed	No	Model for separation prediction	No
Additional dimension/techniques	IMS	CCS verified by standard	No
How was/were the additional dimension(s) used?	For separation of isobaric/isomeric interference in MS1 and MS2 dimensions	Was a model used to predict lipid molecule separation?	No
Lipid Identification Software	Skyline	Nomenclature for intact lipid molecule	Yes
Nomenclature for fragment ions	Yes		

42) PC O[M+H]+ / Lipid quantification

Quantitative	No	Normalization to reference	No
Batch correction	No		

43) PC O[M+Na]+ / Lipid identification

Lipid class	PC O	MS Level for identification	MS^1, MS^2
Identification level	sn Position	MS^1 adduct	[M+Na]+
Isotope correction at MS^1	No	MS^2 adduct	[M+H]+

Fragments for identification

Fragment name
M+Na-HG(PC,184)
M+Na-TMA

-FA2(+H)

Isotope correction at MS^2	No	MS^1 verified by standard	No
MS^2 verified by standard	No	Background check at MS^1	No
Background check at MS^2	No	Did you presume assumptions for identification?	No
Limit of detection	No	RT verified by standard	No
Separation of isobaric/isomeric interferece confirmed	No	Model for separation prediction	No
Additional dimension/techniques	IMS	CCS verified by standard	No
How was/were the additional dimension(s) used?	For separation of isobaric/isomeric interference in MS1 and MS2 dimensions	Was a model used to predict lipid molecule separation?	No
Lipid Identification Software	Skyline	Nomenclature for intact lipid molecule	Yes
Nomenclature for fragment ions	Yes		

43) PC O[M+Na]+ / Lipid quantification

Quantitative	No	Normalization to reference	No
Batch correction	No		

44) PC P[M+H]+ / Lipid identification

Lipid class	PC P	MS Level for identification	MS^1, MS^2
Identification level	sn Position	MS^1 adduct	[M+H]+
Isotope correction at MS^1	No	MS^2 adduct	[M+H]+
Fragments for identification			

Fragment name
-FA1(+H)
-FA2(+H)

Isotope correction at MS^2	No	MS^1 verified by standard	No
MS^2 verified by standard	No	Background check at MS^1	No
Background check at MS^2	No	Did you presume assumptions for identification?	No
Limit of detection	No	RT verified by standard	No
Separation of isobaric/isomeric interferece confirmed	No	Model for separation prediction	No
Additional dimension/techniques	IMS	CCS verified by standard	No
How was/were the additional dimension(s) used?	For separation of isobaric/isomeric interference in MS1 and MS2 dimensions	Was a model used to predict lipid molecule separation?	No
Lipid Identification Software	Skyline	Nomenclature for intact lipid molecule	Yes
Nomenclature for fragment ions	Yes		

44) PC P[M+H]+ / Lipid quantification

Quantitative	No	Normalization to reference	No
Batch correction	No		

45) PC P[M+Na]+ / Lipid identification

Lipid class	PC P	MS Level for identification	MS^1, MS^2
Identification level	sn Position	MS^1 adduct	[M+Na]+
Isotope correction at MS^1	No	MS^2 adduct	[M+H]+

Fragments for identification

Fragment name
M+Na-HG(PC,184)
M+Na-TMA
-FA2(+H)
-HG(PC,184)-FA2(+H)

Isotope correction at MS^2	No	MS^1 verified by standard	No
MS^2 verified by standard	No	Background check at MS^1	No
Background check at MS^2	No	Did you presume assumptions for identification?	No
Limit of detection	No	RT verified by standard	No
Separation of isobaric/isomeric interfeence confirmed	No	Model for separation prediction	No
Additional dimension/techniques	IMS	CCS verified by standard	No
How was/were the additional dimension(s) used?	For separation of isobaric/isomeric interference in MS1 and MS2 dimensions	Was a model used to predict lipid molecule separation?	No
Lipid Identification Software	Skyline	Nomenclature for intact lipid molecule	Yes
Nomenclature for fragment ions	Yes		

45) PC P[M+Na]+ / Lipid quantification

Quantitative	No	Normalization to reference	No
Batch correction	No		

46) PE[M+H]+ / Lipid identification

Lipid class	PE	MS Level for identification	MS^1, MS^2
Identification level	Molecular species level	MS^1 adduct	[M+H]+
Isotope correction at MS^1	No	MS^2 adduct	[M+H]+

Fragments for identification

Fragment name
-HG(PE,141)
FA1(+O)
FA2(+O)

Isotope correction at MS^2	No	MS^1 verified by standard	No
MS^2 verified by standard	No	Background check at MS^1	No
Background check at MS^2	No	Did you presume assumptions for identification?	No
Limit of detection	No	RT verified by standard	No

Separation of isobaric/isomeric interferece confirmed	No	Model for separation prediction	No
Additional dimension/techniques	IMS	CCS verified by standard	No
How was/were the additional dimension(s) used?	For separation of isobaric/isomeric interference in MS1 and MS2 dimensions	Was a model used to predict lipid molecule separation?	No
Lipid Identification Software	Skyline	Nomenclature for intact lipid molecule	Yes
Nomenclature for fragment ions	Yes		

46) PE[M+H]+ / Lipid quantification

Quantitative	No	Normalization to reference	No
Batch correction	No		

47) PE[M+Na]+ / Lipid identification

Lipid class	PE	MS Level for identification	MS^1, MS^2
Identification level	Molecular species level	MS^1 adduct	[M+Na]+
Isotope correction at MS^1	No	MS^2 adduct	[M+H]+

Fragments for identification

Fragment name
M+Na-C2H5N-FA1
M+Na-C2H5N-FA2
M+Na-HG
M+Na-az
-HG(PE,141)

Isotope correction at MS^2	No	MS^1 verified by standard	No
MS^2 verified by standard	No	Background check at MS^1	No
Background check at MS^2	No	Did you presume assumptions for identification?	No
Limit of detection	No	RT verified by standard	No
Separation of isobaric/isomeric interferece confirmed	No	Model for separation prediction	No
Additional dimension/techniques	IMS	CCS verified by standard	No
How was/were the additional dimension(s) used?	For separation of isobaric/isomeric interference in MS1 and MS2 dimensions	Was a model used to predict lipid molecule separation?	No
Lipid Identification Software	Skyline	Nomenclature for intact lipid molecule	Yes
Nomenclature for fragment ions	Yes		

47) PE[M+Na]+ / Lipid quantification

Quantitative	No	Normalization to reference	No
Batch correction	No		

48) SM[M+H]+ / Lipid identification

Lipid class	SM	MS Level for identification	MS^1, MS^2
Identification level	sn Position	MS^1 adduct	[M+H]+
Isotope correction at MS^1	No	MS^2 adduct	[M+H]+

Fragments for identification

Fragment name
LCB(-H3O2)

Isotope correction at MS^2	No	MS^1 verified by standard	No
MS^2 verified by standard	No	Background check at MS^1	No
Background check at MS^2	No	Did you presume assumptions for identification?	No
Limit of detection	No	RT verified by standard	No
Separation of isobaric/isomeric interference confirmed	No	Model for separation prediction	No
Additional dimension/techniques	IMS	CCS verified by standard	No
How was/were the additional dimension(s) used?	For separation of isobaric/isomeric interference in MS1 and MS2 dimensions	Was a model used to predict lipid molecule separation?	No
Lipid Identification Software	Skyline	Nomenclature for intact lipid molecule	Yes
Nomenclature for fragment ions	Yes		

48) SM[M+H]+ / Lipid quantification

Quantitative	No	Normalization to reference	No
Batch correction	No		

49) TG[M+NH4]+ / Lipid identification

Lipid class	TG	MS Level for identification	MS^1, MS^2
Identification level	Molecular species level	MS^1 adduct	[M+NH4]+
Isotope correction at MS^1	No	MS^2 adduct	[M+H]+

Fragments for identification

Fragment name
FA1
FA2
FA3
-FA1(+HO)-(NH3)
-FA2(+HO)-(NH3)
-FA3(+HO)-(NH3)

Isotope correction at MS^2	No	MS^1 verified by standard	No
MS^2 verified by standard	No	Background check at MS^1	No
Background check at MS^2	No	Did you presume assumptions for identification?	No
Limit of detection	No	RT verified by standard	No
Separation of isobaric/isomeric interference confirmed	No	Model for separation prediction	No
Additional dimension/techniques	IMS	CCS verified by standard	No

How was/were the additional dimension(s) used?	For separation of isobaric/isomeric interference in MS1 and MS2 dimensions	Was a model used to predict lipid molecule separation?	No
Lipid Identification Software	Skyline	Nomenclature for intact lipid molecule	Yes
Nomenclature for fragment ions	Yes		

49) TG[M+NH4]+ / Lipid quantification

Quantitative	No	Normalization to reference	No
Batch correction	No		
