# Supplementary material for: Increasing PFAS concentrations in human serum correlate with elevated blood lipid levels
Source: Env Sci Adv. 2026 Feb 16;5(3):885–99. doi: 10.1039/d5va00483g (PMC12921673; doi:10.1039/d5va00483g)
Supplement: VA-005-D5VA00483G-s003 [file VA-005-D5VA00483G-s003.pdf]

Supplementary Information (SI) for Environmental Science: Advances.
This journal is © The Royal Society of Chemistry 2026

Supporting Information

Increasing PFAS Concentrations in Human Serum Correlate with Elevated Blood Lipid Levels

Authors

Ashlee T. Falls[1], Anna K. Boatman[1], Jack P. Ryan[1], Amie M. Solosky[1], James N. Dodds[1], Jessie R. Chappel[3], Allison N. Fry[1], Kaylie I. Kirkwood-Donelson[4], Heather M. Stapleton[2*], Erin S. Baker[1*]

Affiliations

[1]Department of Chemistry, University of North Carolina at Chapel Hill, Chapel Hill, NC, 27514

[2]Nicholas School of the Environment, Duke University, Durham, NC, 27708

[3]Bioinformatics Research Center, Department of Biological Sciences, North Carolina State University, Raleigh, NC 27606

[4]National Institute of Environmental Health Sciences, Durham, NC, 27709

*Corresponding authors(s): Heather M. Stapelton (heather.stapleton@duke.edu) and Erin S. Baker (erinmsb@unc.edu)

Table of Contents

Table of Contents

Chemicals and Reagents...2
Serum PFAS Extraction and LC-MS/MS Methods for Targeted Analyses...2
Serum PFAS Extraction for Suspect Screening Analyses...3
PFAS SSA with LC-IMS-MS...3
Serum Lipid Extraction...5
Lipid LC-IMS-CID-MS/MS Analysis...5
Figures...7
Safety Statement...7
References...7

Chemicals and Reagents

For the experiment performed in the study, a stable isotope-labeled PFAS standard mix (MPFAC-HIF-ES) was obtained from Wellington Laboratories (Guelph, Canada). This mix contained 24 PFAS at concentrations ranging from 250 to 5,000 ng/mL in methanol. This was used as an internal standard in the PFAS suspect screening analysis (SSA). The Standard Reference Material 1957 (SRM 1957, Freeze-Dried Organic Contaminants in Non-Fortified Human Serum) was obtained from the National Institute of Standards and Technology (NIST, Gaithersburg, MD) and was used for quality control in the PFAS SSA. [1] Brain Total Lipid Extract (BTLE) from Avanti Polar Lipids (Alabaster, AL) was used for quality control in the lipid analyses. Optima LC-MS grade methanol, water, acetonitrile, chloroform, and ammonium acetate were obtained from Fisher Scientific (Hampton, NH). For specific catalog numbers of all chemicals, reagents, and equipment, see **Table S1**.

Serum PFAS Extraction and LC-MS/MS Methods for Targeted Analyses

For all chemical reagents, PFAS extraction methods, and LC-MS/MS methods for the PFAS targeted analyses, refer to Hall et al. 2023 (drinking water) and Hoxie et al. 2025 (firefighters). [2, 3]

Serum PFAS Extraction for Suspect Screening Analyses

For the PFAS SSA, 50 µL of serum from each sample was aliquoted into 1.5 mL Low Retention microcentrifuge tubes (Thermo Fisher Scientific, Waltham, MA) and spiked with 5 µL of the PFAS internal standard (MPFAC-HIF-ES). 300 µL of pre-chilled (-20°C) LC-MS grade acetonitrile was added for protein precipitation. Samples were chilled for 30 min at -20°C prior to mixing for 30 s using a vortex mixer (VWR, Thorofare, NJ) and centrifuged for 5 minutes at 12,500 g and 4°C using an Eppendorf Centrifuge 5810 (Hamburg, Germany). 200 µL of the supernatant was transferred to a polypropylene Eppendorf tube and dried down using a SpeedVac vacuum concentrator also from Thermo Fisher Scientific. The samples were then reconstituted in 100 µL of 40:60 LC-MS grade methanol:water with 3 mM ammonium acetate and transferred and stored in 1 mL polypropylene LC autosampler vials with inserts (Agilent Technologies; Santa Clara, CA) at -20°C prior to analysis. Method blanks (50 µL water) and SRM 1957 quality control samples were also extracted with the same process. [1] A standard blank (5 µL MPFAC-HIF-ES and 95 µL of 40:60 methanol:water with 3 mM ammonium acetate) and a double blank (200 µL of 40:60 methanol:water with 3 mM ammonium acetate) were also prepared directly in polypropylene LC autosampler vials for the study.

PFAS SSA with LC-IMS-MS

PFAS SSA were performed using an Agilent 1290 Infinity II UPLC system coupled to an Agilent 6560 IM-QTOF MS platform (Santa Clara, CA) using a method previously developed for PFAS profiling. [4-6] The Agilent ESI-L Low Concentration tune mix solution (Santa Clara, CA) was directly injected in the 6560 IM-QTOF for verification of mass accuracy and collision cross section (CCS) calibration prior to sample analysis. Double blanks, standard blanks, method blanks, and SRM 1957 were injected in triplicate at a volume of 4 µL for chromatographic separation on an Agilent Zorbax Eclipse Plus™ C18 column (Santa Clara, CA) (2.1 mm x 50 mm, 1.8 µm particle size) with an in-line 5 mm guard column. Samples were then injected in a randomized order with a double blank and standard blank injection between every ten sample injections to monitor carryover and instrument performance. Mobile phase A (MPA) consisted

of water with 5 mM ammonium acetate and mobile phase B (MPB) was 95:5 methanol:water with 5 mM ammonium acetate. A 16.5-minute gradient ramping MPB from 10% to 100% was applied at a flow rate of 0.4 mL/min (**Table S2**). Analytes were ionized in negative mode using the Agilent Jet Stream ESI source prior to drift tube IMS separation. Ion packets were pulsed through the drift tube using 4-bit multiplexing. [7] Agilent. d files were collected for each sample, containing the multidimensional LC, IMS, and MS information for further analysis. Source conditions and IMS-MS settings are provided in **Tables S3-4.**

Each data file was demultiplexed using a PNNL PreProcessor (v4.1 2023.06.03) with a signal intensity threshold of 20 counts and 100% pulse coverage. [6, 7] The Agilent ESI tune mix file was used to create a calibration curve for the single-field method and Agilent IM-MS Browser (v10.0) to relate known CCS values to observed drift times. The resulting single-field CCS method function (ß and t_{fix}) was applied to all files to allow previously characterized PFAS using an in-house library of 100 PFAS. [4, 8] All data files were then imported into and evaluated using Skyline (MacCoss Lab Software, v23.1), an open source and vendor neutral software for MS data processing. Parameters including LC retention time index, CCS values within the resolving power window of 30, and *m/z* values with a mass error less than 10 ppm were manually assessed and compared to the library. [4, 9, 10] Peaks were further validated by precursor isotope distribution and retention time alignment to internal standards. Peak areas were normalized to the abundance of their corresponding internal standard when applicable by a light/heavy ($^{12}C/^{13}C$) ratio. When an exact match PFAS standard was not available, a surrogate standard was assigned based on similarities of class, chain length, and retention time. From Skyline, relative PFAS abundances were calculated by subtracting the average of the method blank peak areas from the normalized peak area ratios. The limit of detection (LOD) was set as the average detection in the method blanks plus 3 times the standard deviation. Positive values following the subtraction that were less than the LOD were replaced with the LOD/2 and values that were negative following the subtraction were set to zero. [2] Results were imported into RStudio (v2024.04.2) for a Wilcoxon rank-sum test with Bonferroni corrections for group differences for PFAS not previously targeted and were detected in at least 3 samples in each group. [11, 12]

Serum Lipid Extraction

For lipidomic analyses, 50 µL of serum was aliquoted into 1.7 mL Sorenson SafeSeal™ Microcentrifuge tubes (Waltham, MA) and stored at -80°C. Sample identities were blinded and randomized prior to preparation to control for potential batch effects. A previously validated modified Folch lipid extraction method was applied to the samples.[13, 14] Here, 600 µL of 2:1 cold (-20°C) chloroform/methanol was added to the serum. Each sample was vortexed for 30 s using an analog vortex mixer (VWR; Thorofare, NJ) and 150 µL of water was added prior to mixing again for 30 s. Samples sat for 5 min at room temperature prior to centrifugation at 12,000 g for 10 min at 4°C with an Eppendorf Centrifuge 5810 (Hamburg, Germany) to ensure phase separation. Samples were then placed on ice to prevent the layers from diffusing and degrading.[15] Because this liquid/liquid extraction provides a top (aqueous) and bottom (organic) extraction layer, 100 µL of the organic layer was transferred to a new tube and dried down using a SpeedVac vacuum concentrator (ThermoFisher Scientific; Waltham, MA). Samples were reconstituted in 10 µL of chloroform and 190 µL of LC-MS grade methanol and stored in 2 mL amber glass LC autosampler vials with inserts (Agilent Technologies; Santa Clara, CA) at -20°C for less than 1 week prior to analysis. Method blanks (50 µL water) were prepared in triplicate for each batch by the same method to account for background lipid contamination.[14, 15] A double blank of 95:5 methanol/chloroform was prepared directly in a glass LC autosampler vial. BTLE was used as a quality control sample to monitor instrument performance.

Lipid LC-IMS-CID-MS/MS Analysis

Lipidomic analyses of the 78 serum samples were performed on the previously described LC-IMS-CID-MS platform using a method previously developed for lipids.[8, 16] Double blank and BTLE samples were each injected in triplicate, as well as six method blanks at 10 µL volume onto a Waters Acquity UPLC CSH™ C18 column (Milford, MA) (3.0 mm x 150 mm, 1.7 µm particle size). Samples were assigned blind sample IDs (S1-S78) to prevent bias between lipid and PFAS analyses and were injected at 10 µL in randomized order with additional double blank and BTLE injections between every 13 samples to monitor

carry over and instrument performance. MPA was 60:40 acetonitrile:water with 10 mM ammonium acetate and MPB was 90:10 isopropanol:acetonitrile with 10 mM ammonium acetate. The LC gradient is listed in **Table S5**. Following polarity separations, analytes were then ionized with an Agilent Jet Stream ESI source in negative and positive ionization mode for the IMS-MS analyses with source conditions in **Table S6**. The lipidomics data were acquired using alternating frames between MS^1 and MS/MS, utilizing collision-induced dissociation (CID) ramping (**Table S7**). IMS-MS settings are described in **Table S8**. Agilent .d files were collected containing the LC, IMS, CID, and MS/MS information. The Agilent ESI tune mix file was used to create a calibration curve as previously described to characterize lipids using an in-house library of 877 lipids. [9, 17] All data files were then imported into and evaluated using Skyline. LC retention time index, CCS values, *m/z*, and fragmentation patterns were manually processed. [8, 9] Lipid identifications and their corresponding peak areas were exported from Skyline and $\log_2$ transformed for normalization.

For the assessment of lipid differences due to high PFAS serum levels, all 78 samples were split into categories of ≥20 ng/mL and <20 ng/mL based on the National Academies guidelines previously described. [18] A Mann-Whitney U test was performed with Benjamini-Hochberg corrections to identify statistically significant lipids and fold change comparisons between ≥20 ng/mL and <20 ng/mL exposure groups. Statistical analyses were performed using RStudio (v2024.04.2). Lipid peak areas were also imported into MetaboAnalyst 6.0 for data visualization by principal component analysis (PCA) and hierarchical clustering (based on Euclidean distances and clustering with the Ward method) to observe group separations and lipid abundance trends. [19] All detected lipid features were imported into RStudio (v2024.04.2) with 8 PFAS SSA peak areas (>50% detection frequency) for Spearman correlation tests to assess significant relationships between analytes.

Figures

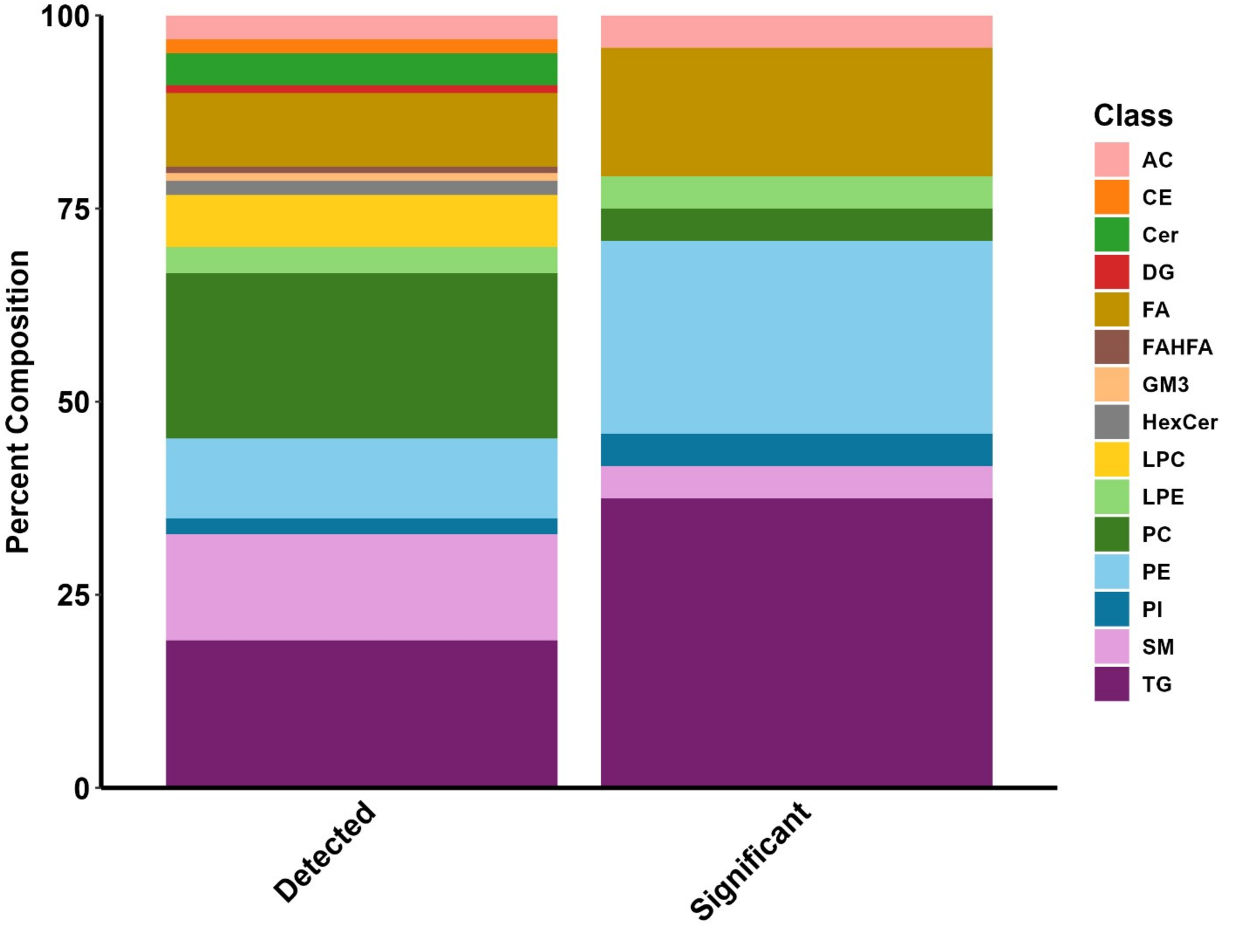

Figure S1. Percent composition of detected lipids by lipid class compared to the composition of significantly altered lipids. Phosphatidylethanolamines (PEs) and triglycerides (TGs) were enriched using fisher's exact test ($p_{adj} < 0.05$).

Safety Statement

No unexpected or unusually high safety hazards were encountered.

References

(1) National Institute of Standards and Technologu. SRM 1957; Organic Contaminants in Non-Fortified Human Serum (Freeze-Dried).

(2) Hall, S. M.; Zhang, S.; Tait, G. H.; Hoffman, K.; Collier, D. N.; Hoppin, J. A.; Stapleton, H. M. PFAS levels in paired drinking water and serum samples collected from an exposed community in Central North Carolina. *Science of The Total Environment* **2023**, *895*, 165091. DOI: https://doi.org/10.1016/j.scitotenv.2023.165091.

(3) Hoxie, T. E. S. Assessing Exposure to Per- and Polyfluoroalkyl Substances in the Indoor and Ambient Environment Utilizing Silicone Wristbands. 2024. https://hdl.handle.net/10161/32628.

(4) Dodds, J. N.; Hopkins, Z. R.; Knappe, D. R. U.; Baker, E. S. Rapid Characterization of Per- and Polyfluoroalkyl Substances (PFAS) by Ion Mobility Spectrometry-Mass Spectrometry (IMS-MS). *Anal Chem* **2020**, *92* (6), 4427-4435. DOI: 10.1021/acs.analchem.9b05364 From NLM.
(5) Kirkwood, K. I.; Fleming, J.; Nguyen, H.; Reif, D. M.; Baker, E. S.; Belcher, S. M. Utilizing Pine Needles to Temporally and Spatially Profile Per- and Polyfluoroalkyl Substances (PFAS). *Environ Sci Technol* **2022**, *56* (6), 3441-3451. DOI: 10.1021/acs.est.1c06483 From NLM.
(6) Boatman, A. K.; Chappel, J. R.; Polera, M. E.; Dodds, J. N.; Belcher, S. M.; Baker, E. S. Assessing Per- and Polyfluoroalkyl Substances in Fish Fillet Using Non-Targeted Analyses. *Environmental Science & Technology* **2024**. DOI: 10.1021/acs.est.4c04299.
(7) Ibrahim, Y. M.; Baker, E. S.; Danielson, W. F., 3rd; Norheim, R. V.; Prior, D. C.; Anderson, G. A.; Belov, M. E.; Smith, R. D. Development of a New Ion Mobility (Quadrupole) Time-of-Flight Mass Spectrometer. *Int J Mass Spectrom* **2015**, *377*, 655-662. DOI: 10.1016/j.ijms.2014.07.034 From NLM.
(8) Stow, S. M.; Causon, T. J.; Zheng, X.; Kurulugama, R. T.; Mairinger, T.; May, J. C.; Rennie, E. E.; Baker, E. S.; Smith, R. D.; McLean, J. A.; et al. An Interlaboratory Evaluation of Drift Tube Ion Mobility-Mass Spectrometry Collision Cross Section Measurements. *Anal Chem* **2017**, *89* (17), 9048-9055. DOI: 10.1021/acs.analchem.7b01729 From NLM.
(9) Kirkwood, K. I.; Christopher, M. W.; Burgess, J. L.; Littau, S. R.; Foster, K.; Richey, K.; Pratt, B. S.; Shulman, N.; Tamura, K.; MacCoss, M. J.; et al. Development and Application of Multidimensional Lipid Libraries to Investigate Lipidomic Dysregulation Related to Smoke Inhalation Injury Severity. *J Proteome Res* **2022**, *21* (1), 232-242. DOI: 10.1021/acs.jproteome.1c00820 From NLM.
(10) Kirkwood, K. I.; Pratt, B. S.; Shulman, N.; Tamura, K.; MacCoss, M. J.; MacLean, B. X.; Baker, E. S. Utilizing Skyline to analyze lipidomics data containing liquid chromatography, ion mobility spectrometry and mass spectrometry dimensions. *Nat Protoc* **2022**, *17* (11), 2415-2430. DOI: 10.1038/s41596-022-00714-6 From NLM.
(11) Marvel, S.; To, K.; Grimm, F.; Wright, F.; Rusyn, I.; Reif, D. ToxPi Graphical User Interface 2.0: Dynamic exploration, visualization, and sharing of integrated data models. *BMC Bioinformatics* **2018**, *19* (1), 80.
(12) Reif, D.; Martin, M.; Tan, S.; Houck, K.; Judson, R.; Richard, A.; Knudsen, T.; Dix, D.; Kavlock, R. Endocrine profiling and prioritization of environmental chemicals using ToxCast data. *Environmental Health Perspectives* **2010**, *118* (12), 1714-1720.
(13) Kirkwood, K. I. Advancing Analytical Techniques to Investigate Per- and Polyfluoroalkyl Substances (PFAS) Exposure and Metabolic Impacts. North Carolina State University, 2023. https://www.lib.ncsu.edu/resolver/1840.20/40800.
(14) Folch, J.; Lees, M.; Sloane Stanley, G. H. A simple method for the isolation and purification of total lipides from animal tissues. *J Biol Chem* **1957**, *226* (1), 497-509. From NLM.
(15) Shang, X.; Du, J.; Zhao, Y.; Tian, J.; Jiang, S. Effect of Multiple Freeze-Thaw Cycles on Lipid Degradation and Lipid Oxidation of Grass Carp Surimi Containing Different Amounts of Pork Back Fat. *Food Sci Anim Resour* **2021**, *41* (6), 923-935. DOI: 10.5851/kosfa.2021.e46 From NLM.
(16) May, J. C.; Goodwin, C. R.; Lareau, N. M.; Leaptrot, K. L.; Morris, C. B.; Kurulugama, R. T.; Mordehai, A.; Klein, C.; Barry, W.; Darland, E.; et al. Conformational ordering of biomolecules in the gas phase: nitrogen collision cross sections measured on a prototype high resolution drift tube ion mobility-mass spectrometer. *Anal Chem* **2014**, *86* (4), 2107-2116. DOI: 10.1021/ac4038448 From NLM.
(17) Kirkwood-Donelson, K. I.; Chappel, J.; Tobin, E.; Dodds, J. N.; Reif, D. M.; DeWitt, J. C.; Baker, E. S. Investigating mouse hepatic lipidome dysregulation following exposure to emerging per- and polyfluoroalkyl substances (PFAS). *Chemosphere* **2024**, *354*, 141654. DOI: https://doi.org/10.1016/j.chemosphere.2024.141654.
(18) National Academies of Sciences Engineering and Medicine (U.S.). Committee on the Guidance on PFAS Testing and Health Outcomes; National Academies of Sciences Engineering and Medicine (U.S.). Board on Population Health and Public Health Practice; National Academies of Sciences Engineering and Medicine (U.S.). Board on Environmental Studies and Toxicology. *Guidance on PFAS exposure, testing, and clinical follow-up*; National Academies Press,, 2022.

(19) Xia, J.; Psychogios, N.; Young, N.; Wishart, D. S. MetaboAnalyst: a web server for metabolomic data analysis and interpretation. *Nucleic Acids Res* **2009**, *37* (Web Server issue), W652-660. DOI: 10.1093/nar/gkp356 From NLM.
